# Acyl-CoA Thioesterase 1 Contributes to Transition of Steatosis to Metabolic-Associated Steatohepatitis

**DOI:** 10.1155/2024/5560676

**Published:** 2024-07-11

**Authors:** Elisa Pasini, Cristina Baciu, Marc Angeli, Bianca Arendt, Diogo Pellegrina, Jüri Reimand, Keyur Patel, George Tomlinson, Mohammad T. Mazhab-Jafari, Lakshmi P. Kotra, Sandra Fischer, Johane P. Allard, Atul Humar, Mamatha Bhat

**Affiliations:** ^1^University Health Network, Toronto, Canada; ^2^Ontario Institute for Cancer Research, Toronto, Canada; ^3^University of Toronto, Toronto, Canada

## Abstract

**Background:**

Metabolic dysfunction-associated steatohepatitis (MASH) has become the leading cause of chronic liver disease, but there has been no approved pharmacotherapy to date.

**Methods:**

We used a network analysis approach to delineate protein-protein interactions that contribute to the transition from steatosis to MASH, in order to identify and target this transition as a potential pharmacotherapeutic strategy. Acyl-CoA thioesterase 1 (ACOT1) was identified as a critical node in the protein-protein interaction (PPI) network of the transition from steatosis to MASH in patient samples. ACOT1 overexpression and silencing effects were tested *in vivo* on C57BL/6 mice exposed to high-fat diet (HFD) and inoculated with an adenoviral system to modulate *ACOT1* expression. Transcriptomic and untargeted lipidomic profiles were performed on the mouse livers.

**Results:**

ACOT1 expression was 3-fold higher in MASH as compared to steatosis. In patient samples, *ACOT1* was significantly correlated with the severity of MASH as reflected by the nonalcoholic fatty liver disease score. Experimental validation showed that downregulation of ACOT1 resulted in decreased lipid accumulation and prevention of MASH *in vivo*. Conversely, upregulation of ACOT1 via an adenoviral vector resulted in development of MASH, whereas control mice only developed steatosis. Lipidomic analysis revealed glycerophospholipids to be especially abundant in MASH accelerated by ACOT1 upregulation.

**Conclusion:**

These results suggest that ACOT1 contributes to the transition from steatosis to MASH through modulation of glycerophospholipid accumulation and its potential as a novel therapeutic target in MASH. This trial is registered with NCT02148471.

## 1. Introduction

Metabolic-associated steatotic liver disease (MASLD) affects up to 30% of the North American population, and 25% of the global population [1]. It encompasses a spectrum of liver disease, progressing from simple steatosis to metabolic dysfunction-associated steatohepatitis (MASH), with subsequent risks of developing cirrhosis with its associated complications, including hepatocellular carcinoma and need for liver transplantation [2].

The progression of MASLD is predicated on multiple hits, starting with dysregulated fat metabolism [3], insulin resistance, and toxic lipid-induced hepatocyte injury (lipotoxicity) [4]. Lipotoxicity leads to a proinflammatory microenvironment, with mitochondrial dysfunction [5, 6], endoplasmic reticulum stress [7, 8], apoptosis [3, 7], and immune response [9] contributing to MASH pathogenesis based principally on data from animal models. Although various genes have been investigated individually through knockdown and upregulation in MASLD, the molecular basis of MASH has not been investigated through the evaluation of betweenness centrality in the protein-protein interaction networks, or *interactome*. Networks are depicted as annotated graphs, delineated by nodes (like genes or proteins) and edges (which denote connections between proteins, such as physical interactions). The centrality of a protein in the interactome can be computed and reflects its importance in disease pathogenesis: nodes with high betweenness centrality lie on communication paths controlling information flow. The most central node therefore potentially represents a critical protein in disease pathogenesis and a potential target for drug discovery [10]. This is a unique approach to identification of key drivers of disease pathogenesis that has not been previously applied to the study of MASH, a disease condition for which there are no currently approved therapies. Additionally, most investigational therapies have targeted the inflammation aspect of MASH rather than the lipid accumulation aspect.

Therefore, our goal was to use a network analysis approach to identify novel regulators of the transition from steatosis to MASH, starting with the analysis of high-throughput gene expression data from patients. Using this approach, we identified acyl-CoA thioesterase 1 (ACOT1), a cytoplasmic thioesterase that converts acyl-CoAs to fatty acids and CoA, as being a central node in the interactome of the transition from steatosis to MASH, with a 3-fold higher expression in MASH patients compared to steatosis. We experimentally upregulated and downmodulated ACOT1 in the livers of mice exposed to a high-fat diet (HFD). Upregulation of ACOT1 resulted in MASH via increased accumulation of glycerophospholipids, as compared to steatosis only in control mice. Conversely, downregulation of ACOT1 prevented the development of MASH in mice, with confirmatory transcriptomic and lipidomic profiles. These results suggest a role for ACOT1 in MASH through modulation of glycerophospholipid accumulation, and its potential as a novel therapeutic target in MASH.

## 2. Methods

### 2.1. Integration of High-Throughput MASLD Data from Gene Expression Omnibus

All available high-throughput microarray gene expression datasets related to MASLD (profiling patients with MASH and simple steatosis (SS)) were retrieved from the Gene Expression Omnibus (GEO) (Table S1) using the following query (transcriptome [All Fields] AND NASH [All Fields]) AND “Homo sapiens”[porgn]. To collect high-throughput array data on C57BL/6 mice exposed to HFD as a model of MASH, we used the following search (“transcriptome AND NASH”) AND “Mus musculus”[porgn: txid10090]. The search was performed prior the change in fatty liver disease nomenclature in 2023. Transgenic mice or MASH generated with the methionine-choline deficient diet were not considered for inclusion.

The datasets selected (GSE57290, GSE59042) were analyzed using GEO2R software (https://www.ncbi.nlm.nih.gov/geo/info/geo2r.html) with GEOquery and limma [11] R packages. The genes with *p* value < 0.05 and expression fold-change (FC) values below ≤0.7 or above ≥1.5 were considered significant and identified as differentially expressed genes (DEGs) between the animals showing a MASH phenotype compared to SS or control mice.

### 2.2. Interactomic Approach to Gene Expression Data in MASLD

**Figure 1:**
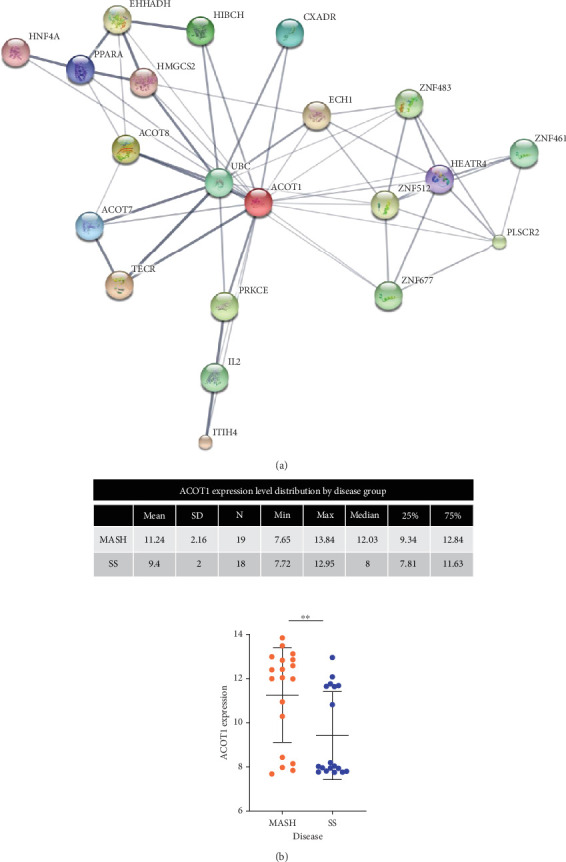
(a) Protein-protein interaction network generated from differentially expressed genes between patients with MASH versus simple steatosis. (b) Scattered dot plots with mean ± SD (⁣^∗∗^*p* value = 0.0050, Mann–Whitney test). *n* = number of samples in each group; SD = standard deviation; SS = simple steatosis.

Gene expression data from patients with simple steatosis (*n* = 20) and MASH (*n* = 22) were retrieved from the dataset GSE89632 available on GEO. The patients were classified based on liver histology, as reported by Arendt et al. [12]. The microarray results were used to perform betweenness centrality analysis [12]. Based on DEGs between groups, the network evolution from steatosis to MASH was investigated following the workflow in Figure S1. The final protein-protein interaction (PPI) network (Figure 1(a)) used for centrality analysis was generated with a combination of tools and databases: IPA software (QIAGEN Inc.), Integrated Interactions Database (IID), and STRING (https://string-db.org/).Tosummarize, 22 genes were differentially expressed between patients with steatohepatitis as compared to steatosis only [12]). These genes were superimposed with genes associated with hepatic steatosis and inflammatory response in IPA (Table S2A). The two genes identified as common, ACOT1 and *SPP1*, were then used as input into the IID to generate further seed genes. Of the seed genes generated, only ACOT1 was found in the list of DEGs and used to generate the PPIs in STRING (PPI enrichment *p* value = 1.46*E*-06) with a maximum of 20 linkers (Table S2B). The output interaction table from STRING was used for calculation of the betweenness centrality score using igraph package [13] (version 1.01) in R [14] (version 3.3.2). For more information on generating this network and statistical tests applied, please refer to the Supporting Information (available here).

### 2.3. Impact of ACOT1 Modulation in a Mouse Model of MASLD

The University Health Network (UHN) animal care and use program is certified by the Canadian Council on Animal Care, and all procedures were approved by the UHN animal care committee (AUP#5620). The animal study was approved by the University Health Network Animal Research Committee and performed in accordance with the relevant guidelines and regulations and reported in accordance with ARRIVE guidelines (https://arriveguidelines.org).

Six- and 8-week-old male C57BL/6 mice were housed in sterilized, individually ventilated cages. After 1 week of acclimation, animals received free access to high-fat, high-carbohydrate diet (HFHC) for 16 weeks [15]. HFHC diets consisted of 58 kcal% fat diet (D12330 high-fat diet from research diets) supplemented with water enriched with 42 g/L carbohydrate at a ratio of 45% sucrose and 55% fructose on a 12 : 12 h light-dark cycle. Humidity was maintained between 40 and 70% and temperature between 20 and 22°C. Animal weight was recorded weekly. Serum glucose and liver profile panel were assessed using the VetScan VS2 platform (Abaxis, USA).

For the animal study of ACOT1 upregulation, the mice (*n* = 10) were divided into 2 groups after 12 weeks on the HFD diet, and tail vein injections of either an adenoviral vector (with adenoviral type 5 backbone) containing Ad-GFP-m-ACOT1 or Ad-GFP at 1 × 10^9^ PFU were performed weekly for 4 weeks (Vector Biolabs). The construct name was Ad-GFP-m-ACOT1, with adenoviral type 5 backbone. The mouse ACOT1 gene was placed with a tag/marker eGFP under the same CMV promoter. Blood and liver tissue were collected at the time of sacrifice (week 16).

For the ACOT1 downmodulation animal study, we awaited the clear confirmation of mild MASH, which occurred by 18 weeks on the HFD diet. At that point, the mice (*n* = 12) were divided in 2 groups, and tail vein injections of either Ad-GFP-U6-scramble-shRNA or Ad-GFP-U6-m-ACOT1-shRNA at 1 × 10^9^ PFU were performed weekly for 6 weeks (Vector Biolabs). Blood and liver tissue were collected at the time of sacrifice (week 25), as described above.

### 2.4. Histological Analysis

Liver histology was assessed using hematoxylin and eosin (H&E) stains in paraffin-embedded sections. Entire slides were digitally scanned by a Leica ScanScope AT2 (brightfield) scanning system (Leica, Germany) at 40x magnification and analyzed by Aperio ImageScope Viewer (Leica, CA) using the Positive Pixel Count v9 algorithm. Masson's trichrome stain was performed to assess the presence of fibrosis. Ballooning and other relevant histological features necessary for a general MASLD scoring in mice in comparison to human liver pathology were obtained as previously described [16] using the Zeiss Axio Imager widefield microscope with a Carl Zeiss Plan Neofluar 100x/1.30 Oil Objective (Carl Zeiss, Germany). The captured images were then visualized with the Zeiss ZEN lite Digital Imaging Software blue 2.6 software. Slides were reviewed by an expert liver pathologist (SF) blinded to the dietary and experimental conditions of the mice.

### 2.5. Gene Expression

Gene expression microarray analysis was performed on livers using the Affymetrix Mouse Gene 2.0 ST platform (Thermo Fisher, Waltham, MA). Raw data was preprocessed by the RMA algorithm using the oligo [17] package. Statistical analysis was performed with limma [11], data annotation with annotate, and pd.mogene.2.0.st packages in R [18, 19]. For differential expression analysis, we compared mice with ACOT1 upregulation (Ad-GFP-m-ACOT1) versus their matched controls (Ad-GFP), whereas in the silencing experiment, we compared Ad-GFP-U6-m-ACOT1-shRNA mice versus Ad-GFP-U6-scramble-shRNA (empty) mice. A gene was considered differentially expressed if *p* value < 0.05 (no FDR correction at this time) and FC ≥ 1.5 or FC ≤ 0.7. For identification of pathways, upstream regulators, and networks, we employed QIAGEN IPA (QIAGEN Inc., https://digitalinsights.qiagen.com/IPA). Gene ontology enrichment analysis was performed using the web tool MGI 6.13 available at http://www.informatics.jax.org/mgihome [20, 21].

### 2.6. Real-Time PCR

RNA was isolated from the mouse livers using the QIAGEN RNeasy mini kit (QIAGEN, Valencia, CA) according to manufacturer's instructions. cDNA was synthesized using the QuantiTect Reverse Transcription Kit (QIAGEN, Valencia, CA). RT-PCR was performed on the Step One Plus platform (Applied Biosystem, Carlsbad, CA) using the SYBR Green Real-Time PCR Master Mix (Thermo Fisher, Waltham, MAD). Data was normalized to the average *C*_T_ value for Hprt for mice or HPRT for HepG2 cells. The primers used are described below:

Acot1-mouse forward: 5⁣′-GATCGCCTCAAGATGTTGT-3⁣′

Acot1-mouse reverse: 5⁣′-ATGATCTGGGGCTTCTCCTT-3⁣′

Hprt-mouse forward: 5⁣′-CCTAAGATGAGCGCAAGTTGAA-3⁣′

Hprt-reverse: 5⁣′-CCACAGGACTAGAACACCTGCTAA-3⁣′

### 2.7. Lipidomic Data Analysis

Flash-frozen liver samples obtained at sacrifice were analyzed by Sciex 6600 Q-TOF high-resolution mass spectrometer at the Analytical Facility for Bioactive Molecules (AFBM), Hospital for Sick Children (Toronto, Canada). Untargeted lipidomic data were acquired in an unbiased fashion and run through a lipid database to generate potential lipid IDs, which were then used to characterize and compare compositional differences between sample sets. Data analysis was performed using MetaboAnalyst 4.0 software [22] (http://www.metaboanalyst.ca/). Data integrity check, processing, quantile normalization, log2 transformation, and autoscaling were carried out prior to analysis. MetaboAnalyst 4.0 provides a variety of statistical methods, fold change, *t*-tests, and volcano plot, as a combination of the first two methods. For both analyses, ACOT1-overexpressed mice vs. controls and ACOT1-silenced mice vs. controls, we performed *t*-test. The features with *p* value (no FDR correction at this time) < 0.05 and twofold change were considered significant between the corresponding groups.

Untargeted lipidomics of the ACOT1-overexpressing mice vs. controls identified 3540 lipids. 1212 features with constant or single value were removed, resulting in a set of 2328 lipids that were normalized and used for *t*-test. Filtering by *p* value and FC reduced the list of significantly changed lipids between the two groups to 66.

Similarly, we started with 1582 lipids in ACOT1-downmodulated mice vs. controls. During the processing step, 639 features were removed; the remaining 943 were normalized. After applying *t*-test with the above filters, 94 lipids were considered significantly altered between the two groups.

### 2.8. ACOT1 Level and Severity of MASH

Deidentified histological patient data were accessed on April 9, 2020. Histological features of MASLD (ballooning, inflammation, and fibrosis) on liver biopsy and correlation with ACOT1 levels were evaluated by a board-certified liver pathologist (SF). Statistical significance of the relationship between these features and ACOT1 expression level was investigated using the Kruskal-Wallis test for difference in medians between groups. The Wilcoxon rank-sum test was performed in case of binary element, such as SS/MASH or 0/1 values for ballooning. The Spearman correlation was used to investigate the significance of correlation between ACOT1 expression in patients and the NAS score.

### 2.9. Statistical Analysis

Data are presented as mean ± SD. Comparison between two groups was performed with two-tailed unpaired Student *t*-test and multiple comparison with one-way ANOVA followed by Tukey's post hoc test (Prism 8; GraphPad Software Inc., La Jolla, CA), unless stated otherwise. More details were included for each analysis performed within the corresponding sections. Additional details are explained in the Supporting Information.

## 3. Results

### 3.1. MASH Datasets on Gene Expression Omnibus

Five high-throughput microarray gene expression datasets investigating gene expression changes in MASH patients compared to SS or healthy controls were identified in the GEO database (Table S1A). However, some of the studies had a low or unbalanced number of patients in the two different groups of interest (MASH and SS) or did not distinguish SS from healthy controls in the “No MASH” patient group. This resulted in the inclusion of only one high-throughput gene expression dataset relevant to our study (GSE89632). The clinical characteristics of the included 19 MASH and 20 SS patients are reported in the original paper [12] and were balanced with respect to age, sex, and ethnicity.

### 3.2. Interactomic Analysis Identifies ACOT1 as Central in MASH

Using a combination of software and PPI databases (as described in Materials and Methods, following the flow in Figure S1, and with additional PPI listed in Table S2A-B), we generated the network that reveals the evolution from steatosis to MASH (Figure 1(a)). ACOT1 was identified as the central node of this network (Table S3). ACOT1 mRNA abundance was significantly greater in MASH patients (ACOT1 mean value = 11.21 ± SD 2.16) as compared to steatosis only (ACOT1 mean value = 9.40 ± SD 2.16) with a fold change of 2.99 (Figure 1(b)). The PPI network was associated with the following KEGG pathways: fatty acid elongation (*q*-value = 1.56*E*-04), biosynthesis of unsaturated fatty acids (*q*-value = 1.56*E*-04), PPAR signaling pathway (*q*-value = 1.56*E*-04), fatty acid metabolic process (*q*-value = 8.18*E*-05), carboxylic acid metabolic process (*q*-value = 1.32*E*-04), acyl-CoA metabolic process (*q*-value = 7.59*E*-04), and lipid metabolic process (*q*-value = 3.36*E*-04), with ACOT1 being involved in all of these.

### 3.3. ACOT1 Overexpression Induces MASH in Mice

**Figure 2:**
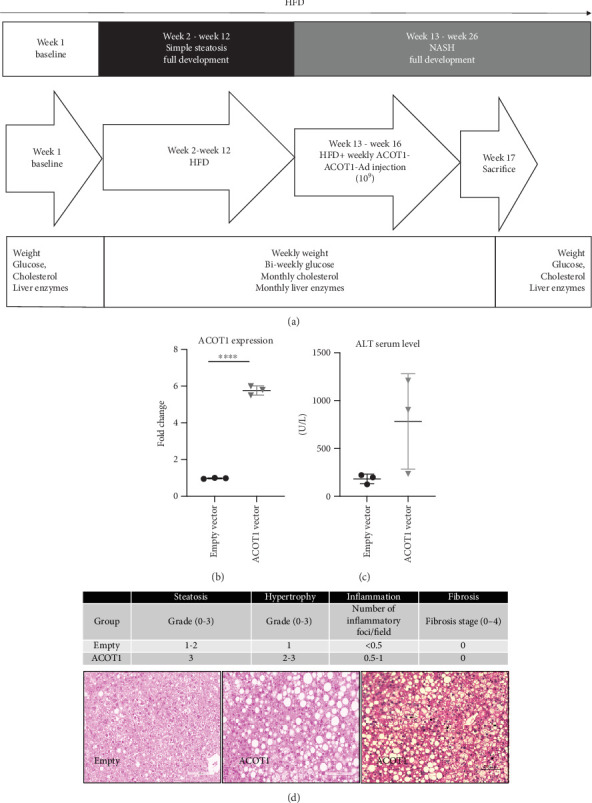
(a) Animal study flowchart: C57BL/6 mouse model exposed to high-fat diet ((58%kcal fat diet) + high sucrose (45%) and high fructose (55%) in water) for 12 weeks, followed by the weekly adenovirus injection to induce overexpression of *ACOT1* continued up to the time of sacrifice at 17 weeks. (b) *ACOT1* gene expression in livers of the mice that received the adenovirus bearing *ACOT1* (*n* = 3) compared to the animal injected with the empty vector (*n* = 3). Scatter plot reports fold-change expression as mean ± SD obtained in each group. ⁣^∗∗∗∗^Student's *t*-test, *p* value < 0.0001. (c) ALT serum level in the two mice groups. Scatter plots reports ALT values as mean ± SD. (d) *ACOT1* gene expression in mice led to hepatocyte ballooning and increased macrovesicular steatosis (grade 3) and ballooning (arrow) in mice overexpressing *ACOT1* gene as compared to control mice (grade 2 steatosis and no evidence of ballooning).

The effect of ACOT1 overexpression was investigated by adenovirus tail vein injection in a HFD MASLD mouse model (Figure 2(a)) starting at 12 weeks of HFD, prior to onset of MASH in the mice. This protocol was followed to determine whether ACOT1 overexpression stimulated development of MASH. At the time of sacrifice (16 weeks), ACOT1 mRNA level was confirmed as significantly increased (*p* value < 0.01) in the animals injected with adenovirus bearing the gene, compared to empty vector (Figure 2(b)). This was accompanied by a relevant increased serum level of alanine aminotransferase (ALT) in the ACOT1 overexpression group versus empty vector (Figure 2(c)).

Histological examination of the liver revealed evidence of ballooning, hypertrophy, and a higher grade (grade 3) of macrovesicular steatosis in the ACOT1-overexpressing mice compared to the control group (Figure 2(d)). The control group had no evidence of histological features of MASH, with presence of only grade 1-2 steatosis. Therefore, ACOT1 overexpression resulted in MASH on histology by 16 weeks, whereas MASH was not seen in control conditions.

Gene expression analysis identified a total of 66 upregulated and 173 downregulated genes between Ad-GFP-m-ACOT1 versus their matched controls (Ad-GFP) (*p* value < 0.05 (no FDR correction at this time) and FC ≥ 1.5 or FC ≤ 0.7). The DEGs were principally involved in lipid metabolism, as well as inflammatory response (Table S4A). Based on the list of affected genes, IPA identified steatosis as the most relevant pathological process related to the liver and defined 5 PPI function-associated networks based on the list of DEGs and their relative molecular partners (Table S4B). All the DEGs are listed in Table S5A.

### 3.4. ACOT1 Silencing Reduces Steatohepatitis *In Vivo*

**Figure 3:**
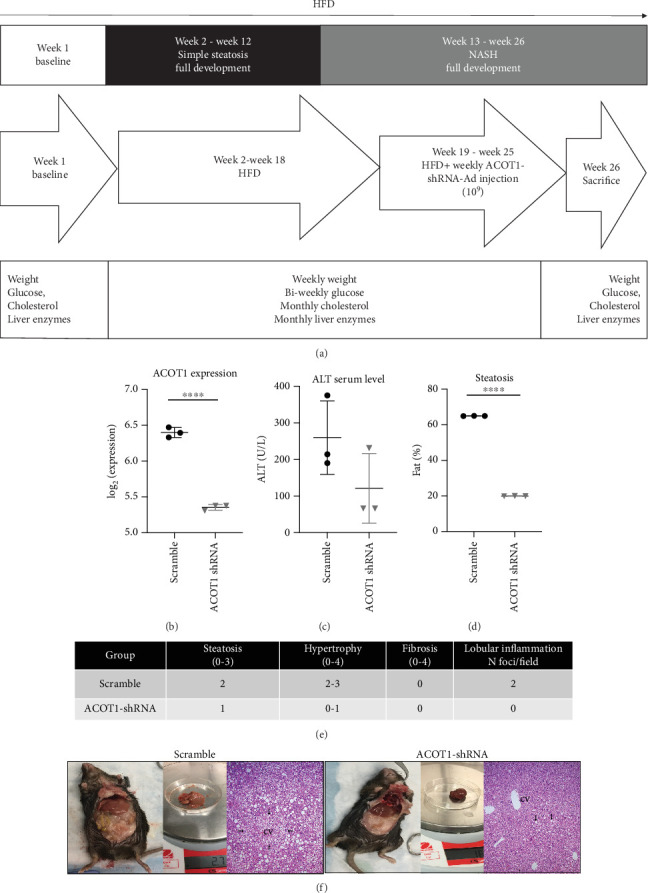
(a) Animal study flowchart: C57BL/6 mouse model exposed to high-fat diet ((58%kcal fat diet) + high sucrose (45%) and high fructose (55%) in water) for 18 weeks before the shRNA adenovirus injection to induce silencing of ACOT1 and was continued up to the sacrifice at 26 weeks. (b) *ACOT1* gene expression in livers of the mice that received the shRNA ACOT1 adenovirus (*n* = 3) compared to the animal injected with the scramble vector (*n* = 3). ⁣^∗∗∗∗^Student's *t*-test, *p* value < 0.0001. (c) ALT serum level in the two mice groups. Scatter plot represents ALT values expressed as mean ± SD obtained in each group. (d) Steatosis was decreased in livers of the mice that received the shRNA-ACOT1 adenovirus (*n* = 3) compared to the animal injected with the scramble vector (*n* = 3). Scatter plot represents ALT values expressed as mean ± SD obtained in each group. ⁣^∗∗∗∗^Student's *t*-test, *p* value < 0.0001. (e) ACOT1 silencing in mice decreased steatosis as well as hypertrophy and inflammation compared to the scramble mice. (f) ACOT1 scramble shows more visceral fat and bigger liver size compared to the shRNA-ACOT1 mice. H&E staining on the scramble mice shows marked microvesicular steatosis in zone 3. Arrows highlight a group of hepatocytes with small fat droplets around a central vein (CV). H&E staining on the shRNA ACOT1 mice livers shows a dramatic decreased of steatosis (magnification 100x).

The effect of ACOT1 silencing was also investigated using the HFD MASLD mouse model (Figure 3(a)). shRNA-ACOT1 weekly injections were started for a period of 6 weeks, once mice had developed MASH by 18 weeks of exposure to HFD (confirmed by elevated ALT). ACOT1-mRNA level was confirmed as significantly decreased in the animals injected with the shRNA-ACOT1 adenovirus compared to the scramble vector (Figure 3(b)) by microarray and was accompanied by a notable decrease in the serum ALT (Figure 3(c)) and a significant decrease in steatosis (*p* = 0.02) (Figure 3(d)). Histological examination of the liver revealed decreased evidence of steatosis, hypertrophy (grade 0-1), and absence of inflammation in shRNA-ACOT1 mice compared to the control group (Figure 3(e)). A visible difference of accumulation of visceral fat, as well as liver weight and size, was noticeable between the two groups of mice (Figure 3(f)). Histological examination of the liver revealed evidence of microvesicular steatosis in zone 3 around a central vein (CV) in the control group as indicated by the arrow in Figure 3(f). Conversely, the shRNA-ACOT1 mice showed a dramatic decrease in steatosis (magnification 100x).

**Figure 4:**
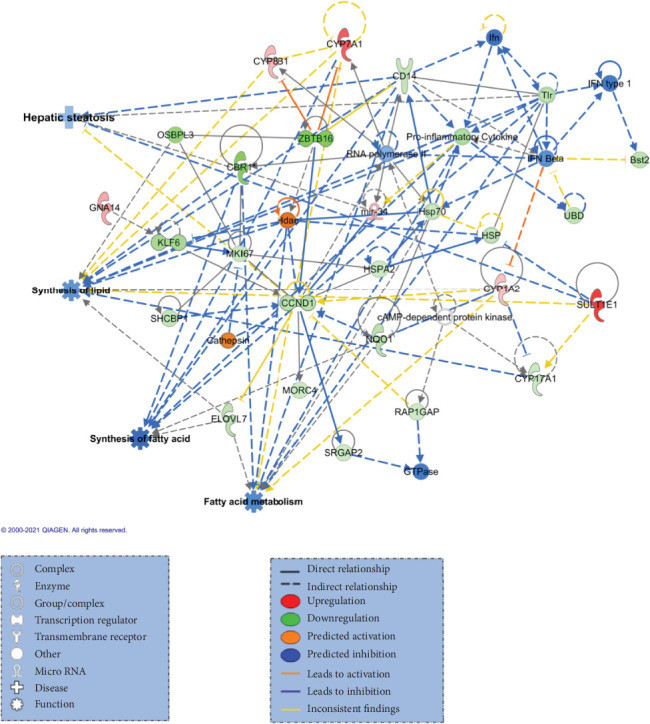
Network 3 (associated with lipid metabolism, small molecule biochemistry), overlapping with diseases and functions in IPA revealed significant enrichment and reduction of hepatic steatosis (*p* value = 7.23*E*-04), synthesis of lipid (*p* value = 1.08*E*-12), synthesis of fatty acid (*p* value = 1.95*E*-06), and fatty acid metabolism (*p* value = 9.085*E*-20).

Gene expression profiling of the mouse liver identified a total of 315 upregulated and 392 downregulated genes (*p* value < 0.05 (no FDR correction at this time) and FC ≥ 1.5 or FC ≤ 0.7) between the two animal groups (Table S5B). Based on this list of DEGs, IPA revealed hepatic fibrosis/hepatic stellate cell activation among the most significant pathways altered (*p* = 3.06*E* − 07) and predicted to be highly inactivated) (Figure S2). Significant alteration was identified for metabolic disease (*p* value range = 1.32*E*-05 to 3.52*E*-21) and gastrointestinal disease (*p* value range = 3.24*E*-05 to 1.03*E*-18). In terms of molecular and cellular functions, lipid metabolism was significantly reduced in shRNA-ACOT1 mice (Table S6). In excellent agreement with the above results, from several networks corresponding to DEGs between the two groups, the lipid metabolism, small molecule biochemistry networks were in the top five networks identified (Table S7). By overlapping network 3 with diseases and functions using IPA Molecule Activity Predictor (MAP) tool, we revealed that hepatic steatosis (*p* value = 7.23*E*-04), synthesis of lipid (*p* value = 1.08*E*-12), synthesis of fatty acid (*p* value = 1.95*E*-06), and fatty acid metabolism (*p* value = 9.085*E*-20) were enriched and significantly reduced (Figure 4), with a *z*-score ranging from -1.91 to -3.05 (Table S6).

### 3.5. Lipidomic Analysis Revealed ACOT1 Modulation Effects on Glycerophospholipid Accumulation *In Vivo*

Untargeted lipidomics of the *ACOT1*-overexpressing mice identified 66 differentially abundant lipids compared to the control mice. Among the differentially abundant lipids, 59/66 were glycerophospholipids, followed by sphingolipids (6/66) and only one 1sterol lipid (1/66) (Table 1). The silencing of ACOT1 in shRNA-ACOT1 mice compared to their matched control mice showed 94 differentially abundant lipids, about 80% of which were glycerophospholipids (75/94) (Table 2). The ACOT1-overexpressing mice had significantly increased accumulation of glycerophospholipids compared to their controls (Table S8).

### 3.6. ACOT1 Gene Expression Level Correlates with Severity of Steatohepatitis

**Figure 5:**
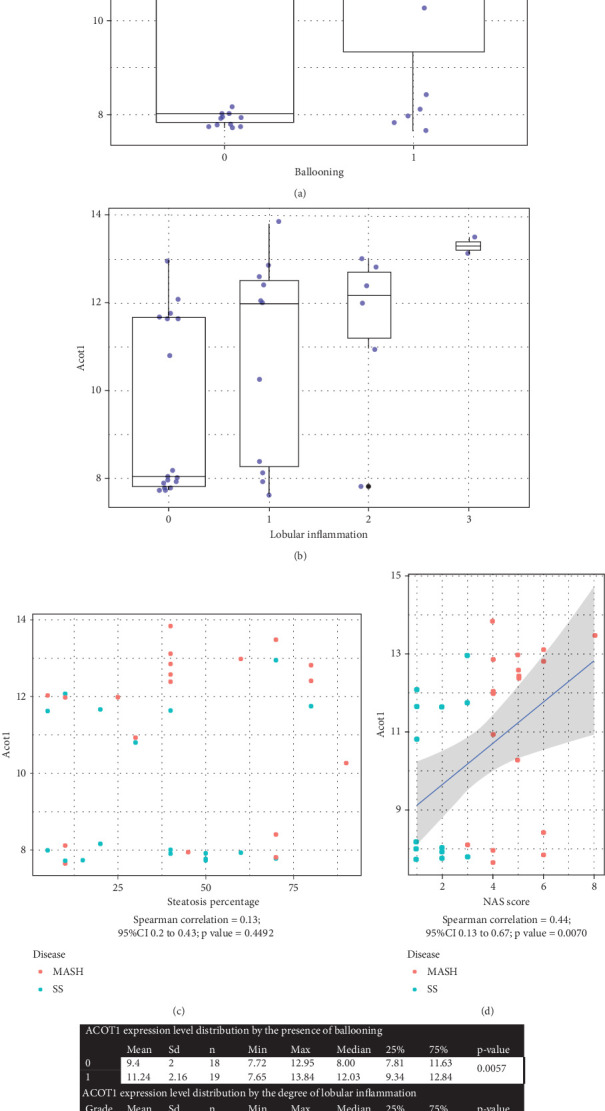
(a) Boxplots show the relationship between *ACOT1* expression and the presence of ballooning, with data summarized in panel (e). Wilcoxon rank-sum test was performed for difference in medians between groups. (b) Boxplots show the relationship between *ACOT1* expression and degree of lobular inflammation, with data summarized in panel (e). Kruskal-Wallis test was performed for difference in medians between groups. (c) *ACOT1* expression corresponding to steatosis percent. *p* value was obtained by the Kruskal-Wallis test. (d) *ACOT1* expression was significantly correlated with MASLD severity score (NAS score) with *p* value < 0.01 (Kruskal-Wallis test). (e) *ACOT1* expression level distribution by the severity of MASH. Wilcoxon rank-sum test was performed for difference in the median of *ACOT1* expression level by disease group and ballooning. Kruskal-Wallis test for difference in the median of *ACOT1* expression level by disease group and the degree of lobular inflammation. SD = standard deviation; *n* = number of samples.

To investigate the relationship between ACOT1 and severity of MASH, we evaluated the correlation between ACOT1 gene expression level and histological features on biopsy (Figures 5(a), 5(b), 5(c), 5(d), and 5(e)). With respect to severity of MASH, ACOT1 mRNA abundance correlated with the presence of ballooning (*p* value = 0.057) and lobular inflammation (*p* value = 0.0153). Importantly, ACOT1 abundance was highly correlated with the MASH score (Spearman correlation = 0.44, 95% CI 0.13 to 0.67, Kruskal-Wallis test *p* value = 0.0070) (Figure 5(d)). Therefore, ACOT1 gene expression level correlated with the severity of MASH in patient samples (Figures 5(d) and 5(e)).

## 4. Discussion

In this study, we used an unbiased network analysis approach to identify ACOT1 as a novel important player in driving MASH. We determined ACOT1 to be central to the protein-protein interaction network (interactome) using the betweenness centrality algorithm and confirmed its contribution to MASH pathogenesis using in vivo and in vitro approaches. ACOT1 overexpression led to MASH, with significantly increased accumulation of glycerophospholipids, whereas the changes of MASH were not seen in the control mice on a high-fat diet. ACOT1 inhibition prevented the development of MASH, along with decreased glycerophospholipid accumulation.

For *in vivo* validation, we generated a mouse model wherein ACOT1 was increased through adenovirus-mediated ACOT1 upregulation along with exposure to a high-fat diet. This supported the importance of ACOT1 in triggering critical lipid accumulation, specifically increasing accumulation of glycerophospholipids and sphingolipids, as evidenced by lipidomics. Increased ACOT1 resulted in de novo lipogenesis with increased macrovesicular hepatic steatosis, along with a gene expression profile compatible with hepatic steatosis and damage. Among the genes involved in lipid metabolism and biosynthesis, *Ctp1a*, *Dhcr24*, and *Pdk4* have been previously reported as upregulated in MASH patients or animal models [23–25]. The overexpression or activation of proinflammatory molecules, such as *Ilrl1*, *Socs2*, *Thbs1*, and *Egfr*, was also aligned with previously published results in MASH [9, 26–28].

Conversely, downregulation of ACOT1 in vivo prevented development of MASH. There was significantly less accumulation of steatosis even with exposure to lipid. Glycerophospholipid accumulation was significantly reduced with ACOT1 inhibition.

We then performed patient-level validation and discovered that there was correlation of ACOT1 with the severity of MASH. Increased ACOT1 expression correlated with hepatocyte ballooning, lobular inflammation, and the MASH activity score, as reflective of the severity of steatohepatitis. There is a rationale for these findings, given that ACOT1 converts acyl-CoAs to free fatty acids (FFA) and CoA, resulting in the critical accumulation of FFAs and subsequent lipotoxicity.

There has been little previous literature on ACOT1, with its biochemistry and structural conformation not having been delineated. A previous study investigated the effect of ACOT1 on fatty acid flux during fasting [29]. In this study, mice were also exposed to a high-fat diet for 12 weeks. However, a knockdown of ACOT1 was performed with only a single injection of adenovirus-mediated targeted knockdown of ACOT1. Mice were then sacrificed when fasting, one week after the injection. With this different experimental approach, hepatic ACOT1 knockdown assessed under fasting conditions was found to decrease liver triglyceride accumulation, by increasing turnover of triglycerides and reducing oxidation of fatty acids. The authors therefore determined that ACOT1 regulates fasting hepatic fatty acid metabolism by balancing oxidative flux and capacity [29]. In contrast, we investigated the effect of persistent ACOT1 upregulation over a month on the liver, while on a high-fat, high-carbohydrate diet. We discovered that this sustained upregulation in ACOT1 resulted in development of steatohepatitis (which did not arise in our control mice at the same time point), with lipidomic analysis demonstrating significantly increased accumulation of glycerophospholipids and sphingolipids.

Accumulation of glycerophospholipids, specifically phosphatidylserine, phosphatidylcholine, phosphatidylethanolamine, and phosphatidylinositol species, has previously been reported in association with greater severity of MASLD [30]. Recent efforts have been made to therapeutically target hepatic lipid accumulation in MASLD [31–33]. For example, acetyl CoA carboxylase inhibition has been found to decrease de novo hepatic lipogenesis and consequent steatosis, although this was associated with a concomitant increase in serum triglycerides. More recently, ACOT9, a mitochondrial enzyme of the ACOT family, was found to modulate hepatic steatosis. However, ACOT9 rescue resulted in steatosis but not the development of MASH. This effect was found to be mediated through triglyceride synthesis and hepatic glucose production [34].

Our search for gene expression datasets was limited by the small number of publicly available datasets profiling MASH patients, often including a small or unbalanced number of patients between MASH and SS and incomplete clinical annotation. We were therefore left with only one eligible dataset for analysis as a starting point, to compare MASH to steatosis. We were more interested in using an unbiased interactomic approach to understand the potential drivers of the transition from steatosis to MASH. Our animal experiment was limited by cessation of the experiment prior to development of hepatic fibrosis, and we therefore could not evaluate the effect of ACOT1 overexpression on development of fibrosis.

## 5. Conclusions

We have shown that ACOT1 contributes to MASH development. Inhibition of ACOT1 resulted in prevention of MASH through decreased accumulation of glycerophospholipids.

Our results suggest ACOT1 as a novel potential therapeutic target, wherein lipid accumulation could be targeted to prevent development of MASH.

## Figures and Tables

**Table 1:** List of the 66 lipids identified as differently abundant between ACOT1-overexpressing mice and the control group.

ACOT1 vector vs. control	FC	*p* value
*Glycerophospholipids*		
PG 440_glycerophospholipid species profile—negative mode	5.4158	0.00083483
PS 422_glycerophospholipid species profile—positive mode	0.18828	0.0019457
DMPE 343_glycerophospholipid species profile—negative mode	10.046	0.0029643
CL 8616_glycerophospholipid species profile—negative mode	0.49116	0.0029878
PE O-460_glycerophospholipid species profile—positive mode	0.39283	0.0033365
PE 467_glycerophospholipid species profile—positive mode	0.39283	0.0033365
141_glycerophospholipid fatty acid profile—negative mode	0.31947	0.0039827
DMPE 342_glycerophospholipid species profile—negative mode	4.6398	0.0047027
PS 362_glycerophospholipid species profile—negative mode	3.6119	0.0048064
121_glycerophospholipid fatty acid profile—positive mode	0.23855	0.0057462
PC O-365AcO_glycerophospholipid species profile—negative mode	4.1669	0.0072979
PE 444_glycerophospholipid species profile—positive mode	14.753	0.0088069
LPE 180_glycerophospholipid species profile—negative mode	2.5034	0.012288
CL 847_glycerophospholipid species profile—negative mode	2.3018	0.012469
CL 7813_glycerophospholipid species profile—negative mode	0.2263	0.012725
PS 374_glycerophospholipid species profile—negative mode	0.23238	0.0139
80_glycerophospholipid fatty acid profile—positive mode	0.18352	0.015693
DMPE O-242_glycerophospholipid species profile—negative mode	0.47585	0.015796
LPI 305_glycerophospholipid species profile—positive mode	0.31311	0.017353
CL 769_glycerophospholipid species profile—negative mode	4.3799	0.018536
MMPE 361_glycerophospholipid species profile—positive mode	6.0512	0.020478
120_glycerophospholipid fatty acid profile—positive mode	0.29508	0.021354
DMPE 385_glycerophospholipid species profile—negative mode	2.0737	0.024004
PC 372AcO_glycerophospholipid species profile—negative mode	6.5025	0.024393
CL 767_glycerophospholipid species profile—negative mode	4.8852	0.026473
LPE 181_glycerophospholipid species profile—negative mode	3.4599	0.028041
DMPE 387_glycerophospholipid species profile—negative mode	2.5637	0.029957
MMPE 364_glycerophospholipid species profile—negative mode	4.948	0.033181
CL 8017_glycerophospholipid species profile—negative mode	3.9143	0.036294
PA 461_glycerophospholipid species profile—negative mode	5.8479	0.039186
MMPE 406_glycerophospholipid species profile—negative mode	2.5567	0.040545
PE 428_glycerophospholipid species profile—positive mode	2.4504	0.044386
PE O-421_glycerophospholipid species profile—positive mode	2.4504	0.044386
PE 387_glycerophospholipid species profile—negative mode	2.6733	0.048282
PS 3811_glycerophospholipid species profile—negative mode	0.10735	0.003149
LPS O-161_glycerophospholipid species profile—negative mode	5.2567	0.0041092
MMPE 408_glycerophospholipid species profile—negative mode	2.2046	0.0048555
LPE 160_glycerophospholipid species profile—negative mode	2.8533	0.006396
CL 8413_glycerophospholipid species profile—negative mode	2.2912	0.010136
PC 366AcO_glycerophospholipid species profile—negative mode	4.1762	0.013525
NAPE 465_glycerophospholipid species profile—negative mode	0.42551	0.013763
LDMPE 242_glycerophospholipid species profile—negative mode	0.47585	0.015796
NAPE 432NH4_glycerophospholipid species profile—positive mode	0.016936	0.017846
DMPE O-380_glycerophospholipid species profile—negative mode	6.4904	0.020186
PC 462_glycerophospholipid species profile—positive mode	4.8021	0.022259
PI 306_glycerophospholipid species profile—positive mode	4.3584	0.023263
CL 502_glycerophospholipid species profile—negative mode	0.11953	0.027643
221_glycerophospholipid fatty acid profile—positive mode	0.11836	0.028334
DMPE 380_glycerophospholipid species profile—negative mode	3.544	0.028633
PS O-387_glycerophospholipid species profile—negative mode	2.3581	0.033074
NAPE 467_glycerophospholipid species profile—negative mode	4.0524	0.03454
NAPE 4714NH4_glycerophospholipid species profile—positive mode	0.20715	0.034867
CL 8617_glycerophospholipid species profile—negative mode	2.2575	0.038586
PG 365_glycerophospholipid species profile—positive mode	3.6213	0.042554
CL 4020_glycerophospholipid species profile—negative mode	6.5606	0.042649
PC O-280AcO_glycerophospholipid species profile—negative mode	4.4949	0.042762
NAPE 380NH4_glycerophospholipid species profile—positive mode	0.18402	0.044394
DMPE 205_glycerophospholipid species profile—negative mode	4.0114	0.046977
MMPE 365_glycerophospholipid species profile—negative mode	2.8672	0.048152
*Sphingolipids*		
MIPC 2702_sphingolipid species profile—positive mode	0.24658	0.0017179
GM2 4742_sphingolipid species profile—positive mode	6.4464	0.016187
SM 3512AcO_sphingolipid species profile—negative mode	2.9097	0.043925
IPC 4032_sphingolipid species profile—negative mode	2.549	0.030693
CerP_sphingolipid class profile—positive mode	0.38165	0.034236
CerP 4742_sphingolipid species profile—positive mode	0.3824	0.041716
*Sterol lipids*		
CE 310NH4_sterol lipid species profile—positive mode	5.0507	2.3365

FC = fold change. *p* value was calculated with two-tailed unpaired Student *t*-test.

**Table 2:** List of the 94 lipids identified as differently abundant between shRNA-ACOT1 mice and the control group.

sh-RNA-ACOT1 vs. control	FC	*p* value
*Glycerophospholipids*		
100_glycerophospholipid fatty acid profile—negative mode	2.9118	0.041139
120_glycerophospholipid fatty acid profile—negative mode	4.116	0.0034868
122_glycerophospholipid fatty acid profile—negative mode	3.3226	0.0022016
226_glycerophospholipid fatty acid profile—negative mode	0.23984	0.016591
CL 724_glycerophospholipid species profile—negative mode	11.573	0.023134
CL 746_glycerophospholipid species profile—negative mode	2.5931	0.041039
CL 7812_glycerophospholipid species profile—negative mode	2.7305	0.010953
CL 788_glycerophospholipid species profile—negative mode	2.7995	0.016176
CL 805_glycerophospholipid species profile—negative mode	2.9058	0.014326
CL 8210_glycerophospholipid species profile—negative mode	2.4697	0.027092
CL 8212_glycerophospholipid species profile—negative mode	3.5823	0.0057443
CL 828_glycerophospholipid species profile—negative mode	5.7078	0.0011763
CL 841_glycerophospholipid species profile—negative mode	10.371	0.0032339
CL 884_glycerophospholipid species profile—negative mode	5.2798	0.017416
DMPE 386_glycerophospholipid species profile—positive mode	0.43699	0.019894
DMPE 406_glycerophospholipid species profile—positive mode	0.36382	0.019765
DMPE 441_glycerophospholipid species profile—negative mode	2.2982	0.039853
DMPE_glycerophospholipid class profile—positive mode	0.41813	0.0074815
LPI 286_glycerophospholipid species profile—negative mode	10.471	0.019241
LPIP 282_glycerophospholipid species profile—negative mode	4.5669	0.013379
LPIP3 140_glycerophospholipid species profile—negative mode	0.18265	0.0044966
NAPE 3512NH4_glycerophospholipid species profile—positive mode	0.20893	5.85*E*-05
NAPE 369NH4_glycerophospholipid species profile—positive mode	0.32624	0.009359
NAPE 380NH4_glycerophospholipid species profile—positive mode	0.0431	0.0082559
NAPE 400NH4_glycerophospholipid species profile—positive mode	0.15838	0.0061374
NAPE 4012NH4_glycerophospholipid species profile—positive mode	0.25225	0.012754
NAPE 446NH4_glycerophospholipid species profile—positive mode	5.3193	0.0052773
NAPE_glycerophospholipid class profile—positive mode	0.21633	0.0051334
PA 420_glycerophospholipid species profile—negative mode	2.1207	0.0014948
PA 467AcO_glycerophospholipid species profile—negative mode	2.8842	0.022741
PA O-4212_glycerophospholipid species profile—negative mode	2.3836	0.035434
PC 321_glycerophospholipid species profile—positive mode	0.20731	0.0013991
PC 342_glycerophospholipid species profile—positive mode	0.4296	0.0033941
PC 345_glycerophospholipid species profile—positive mode	0.20132	0.0027972
PC 405_glycerophospholipid species profile—positive mode	0.15701	0.012474
PC O-366_glycerophospholipid species profile—positive mode	0.21391	0.015842
PC O-424_glycerophospholipid species profile—positive mode	0.34325	0.02618
PC O-426_glycerophospholipid species profile—positive mode	0.46327	0.022478
PC O-466_glycerophospholipid species profile—positive mode	0.056284	0.0018447
PC O-486_glycerophospholipid species profile—positive mode	0.18894	0.0040919
PE 364_glycerophospholipid species profile—positive mode	0.21844	9.56*E*-07
PE 386_glycerophospholipid species profile—positive mode	0.32709	0.017022
PE 426_glycerophospholipid species profile—negative mode	0.39127	0.016858
PE O-361_glycerophospholipid species profile—positive mode	0.14748	0.0099463
PE O-380_glycerophospholipid species profile—positive mode	0.20399	4.33*E*-05
PE O-385_glycerophospholipid species profile—positive mode	0.20375	0.011166
PE O-400_glycerophospholipid species profile—positive mode	0.22382	0.0011385
PE O-445_glycerophospholipid species profile—positive mode	0.16092	0.0089894
PE_glycerophospholipid class profile—positive mode	0.43718	0.0010285
PG 420_glycerophospholipid species profile—negative mode	8.8773	0.0083794
PI 304_glycerophospholipid species profile—negative mode	3.442	0.039452
PI O-301_glycerophospholipid species profile—negative mode	5.4423	0.015903
PI O-323_glycerophospholipid species profile—negative mode	8.8676	0.013429
PIP2 301_glycerophospholipid species profile—negative mode	2.9381	0.032184
PIP3_glycerophospholipid class profile—negative mode	2.5782	0.016807
PS 341_glycerophospholipid species profile—positive mode	0.21346	0.0012066
PS 361_glycerophospholipid species profile—positive mode	0.2236	0.013386
PS 365_glycerophospholipid species profile—positive mode	0.23536	0.0071676
PS 401_glycerophospholipid species profile—negative mode	0.098227	0.0082497
PS 405_glycerophospholipid species profile—negative mode	11.426	0.013146
PS 406_glycerophospholipid species profile—positive mode	0.21562	9.72*E*-07
PS 447_glycerophospholipid species profile—negative mode	2.294	0.041305
*Glycerolipids*		
205_glycerolipid fatty acid profile—positive mode	0.40403	0.0011101
226_glycerolipid fatty acid profile—positive mode	0.1328	0.041733
TAG_glycerolipid class profile—positive mode	2.3609	0.015922
181_glycerolipid fatty acid profile—positive mode	2.3441	0.019353
162_glycerolipid fatty acid profile—positive mode	2.3179	0.017261
160_glycerolipid fatty acid profile—positive mode	2.2481	0.031931
MADAG_glycerolipid class profile—positive mode	2.0553	0.032748
161_glycerolipid fatty acid profile—positive mode	2.05	0.038088
202_glycerolipid fatty acid profile—positive mode	2.3401	0.048118
201_glycerolipid fatty acid profile—positive mode	2.3144	0.023084
*Sphingolipids*		
SM 3912AcO_sphingolipid species profile—negative mode	0.32089	0.012443
GD2 4222_sphingolipid species profile—positive mode	7.1229	0.00099144
HexCer 4022_sphingolipid species profile—positive mode	3.5417	0.0077088
IPC 2942_sphingolipid species profile—negative mode	4.745	0.009337
GD2_sphingolipid class profile—positive mode	5.9716	0.010775
GM1 3232_sphingolipid species profile—negative mode	2.6215	0.01248
GD2 4242_sphingolipid species profile—positive mode	3.2177	0.034576
SGalCer 3512_sphingolipid species profile—negative mode	2.153	0.043
GM1 3242_sphingolipid species profile—negative mode	2.5439	0.046765

FC = fold change. *p* value was calculated with two-tailed unpaired Student *t*-test.

## Data Availability

All data associated with this study are present in the paper or in Supplementary Material. Transcriptomic raw data for ACOT1-overexpressed or ACOT1-silenced mice were deposited in NCBI GEO with accession numbers GSE174797 (https://0-www-ncbi-nlm-nih-gov.brum.beds.ac.uk/geo/query/acc.cgi?acc=GSE174797) and GSE175426 (https://0-www-ncbi-nlm-nih-gov.brum.beds.ac.uk/geo/query/acc.cgi?acc=GSE175426), respectively. Source codes used for gene expression analysis are deposited in GitHub (https://github.com/Diogopell/ACOT1).

## Supplementary Materials

Supplementary Materials Supplemental Materials and Methods: interactomic approach to gene expression data in NAFLD. Table S1: MASH datasets available in GEO. Table S2 (A): list of molecules associated with hepatic steatosis and inflammatory response from diseases and functions table obtained in IPA. The molecules in bold were reported as dysregulated genes in MASH versus SS comparison. Table S2 (B): list of the 58 PPIs obtained by STRING Network analysis. Table S3: betweenness centrality score for PPI network in Figure 1(a). Table S4 (A): list of identified upregulated genes between ACOT1-overexpressing mice and controls, related to metabolism and inflammation. GO = Gene Ontology. PPAR gene targets are in italics. Genes commonly upregulated in two independent mouse datasets (GSE57290, GSE59042) are underlined. Table S4 (B): IPA associated the dysregulated genes in ACOT1-overexpressing mice with steatosis and identified 5 top function-related networks based on the list of affected genes and their molecular interactors. Table S5 (A): list of the DEGs identified between ACOT1-overexpressed mice and the control group. Table S5 (B): list of the DEGs identified between ACOT1-silenced mice and the control group. Table S6: lipid metabolism is inhibited in shRNA-ACOT1 mice, as identified by microarray analysis. In IPA, a *z*‐score ≤ −2 corresponds to a highly inhibited process (shown in bold). Table S7: networks corresponding to DEGs between shRNA-ACOT1 mice and controls. Networks related to lipid metabolism are shown in bold. Table S8: differentially abundant lipids between ACOT1-overexpressing mice and ACOT1 shRNA mice. *n* = number of lipids. Figure S1: workflow for analysis of network evolution from steatosis to MASH. DEGs = differentially expressed genes. Figure S2: canonical pathways significantly dysregulated as identified by IPA from shRNA-ACOT1 mice (*n* = 3) vs. control group (*n* = 3) comparison.
