# Supplementary material for: Acyl-CoA Thioesterase 1 Contributes to Transition of Steatosis to Metabolic-Associated Steatohepatitis
Source: Int J Hepatol. 2024 Jul 11;2024:5560676. doi: 10.1155/2024/5560676 (PMC11611406; doi:10.1155/2024/5560676)
Supplement: Supplementary Materials — Supplemental Materials and Methods: interactomic approach to gene expression data in NAFLD. Table S1: MASH datasets available in GEO. Table S2 (A): list of molecules associated with hepatic steatosis and inflammatory response from diseases and functions table obtained in IPA. The molecules in bold were reported as dysregulated genes in MASH versus SS comparison. Table S2 (B): list of the 58 PPIs obtained by STRING Network analysis. Table S3: betweenness centrality score for PPI network in Figure 1(a). Table S4 (A): list of identified upregulated genes between ACOT1-overexpressing mice and controls, related to metabolism and inflammation. GO = Gene Ontology. PPAR gene targets are in italics. Genes commonly upregulated in two independent mouse datasets (GSE57290, GSE59042) are underlined. Table S4 (B): IPA associated the dysregulated genes in ACOT1-overexpressing mice with steatosis and identified 5 top function-related networks based on the list of affected genes and their molecular interactors. Table S5 (A): list of the DEGs identified between ACOT1-overexpressed mice and the control group. Table S5 (B): list of the DEGs identified between ACOT1-silenced mice and the control group. Table S6: lipid metabolism is inhibited in shRNA-ACOT1 mice, as identified by microarray analysis. In IPA, a z‐score ≤ −2 corresponds to a highly inhibited process (shown in bold). Table S7: networks corresponding to DEGs between shRNA-ACOT1 mice and controls. Networks related to lipid metabolism are shown in bold. Table S8: differentially abundant lipids between ACOT1-overexpressing mice and ACOT1 shRNA mice. n = number of lipids. Figure S1: workflow for analysis of network evolution from steatosis to MASH. DEGs = differentially expressed genes. Figure S2: canonical pathways significantly dysregulated as identified by IPA from shRNA-ACOT1 mice (n = 3) vs. control group (n = 3) comparison. [file 5560676.f1.pdf]

Supplemental Information

Supplemental Materials and Methods

Interactomics approach to Gene Expression data in NAFLD

To generate the final network (**Figure 1A**) that reveals the evolution from steatosis to NASH, we used a series of software and databases, as described below.

We employed IPA software (QIAGEN Inc.) for initial network analysis. IPA identifies the canonical pathways, diseases and functions associated with input data, upstream regulators and biological networks, based on the internal, manually curated Knowledge Base of published experimental findings. Ranking of networks is based on statistical p-value determined by a right-tailed Fisher's exact test(1) and a score that takes into consideration the number of input molecules in context of the total number of entities that can form a specific network. Since the number of input genes was smaller than the IPA requirement for a comprehensive analysis, we performed the intersection of the most biologically meaningful networks and overlaid the results with hepatic steatosis (HS) and inflammatory response (IR) from IPA. The common genes from both HS and IR from each comparison were considered for the next step in our analysis: generating more seeds using Integrated Interactions Database (IID), where we filtered for the liver-specific PPIs based only on experimental evidence. These were then intersected with differentially expressed genes from each group comparison, and the resulting gene lists were inputted individually to STRING (version 10.0), a protein-protein interaction (PPI) software (https://string-db.org/) to create the biological network with a choice of none or multiple interactors on one or two layers. STRING also can provide the statistically significant (FDR)(2) (corrected p-value or q-value) associated KEGG pathways(3-5), biological processes, molecular function, cellular component annotations, etc.

References

1. Fisher RA. On the Interpretation of $\chi 2$ from Contingency Tables, and the Calculation of P. Journal of the Royal Statistical Society,. 1922;85(1):87-94.

2. Benjamini Y, Hochberg Y. Controlling the False Discovery Rate: A Practical and Powerful Approach to Multiple Testing. Journal of the Royal Statistical Society: Series B (Methodological). 1995;57(1):289-300.

3. Kanehisa M, Furumichi M, Tanabe M, Sato Y, Morishima K. KEGG: new perspectives on genomes, pathways, diseases and drugs. Nucleic Acids Research. 2017;45(Database issue):D353-D61.

4. Kanehisa M, Sato Y, Kawashima M, Furumichi M, Tanabe M. KEGG as a reference resource for gene and protein annotation. Nucleic Acids Research. 2016;44(Database issue):D457-D62.

5. Kanehisa M, Goto S. KEGG: Kyoto Encyclopedia of Genes and Genomes. Nucleic Acids Research. 2000;28(1):27-30.

Supplemental Tables

Table S1. MASH datasets available in GEO.

GEO Human	Patients' description	PMID	MASH vs SS (NAFLD)	Comment
GSE66676	Adolescent obese patients undergoing bariatric surgery	26026390	2 defined MASH vs 26 MAFLD	Unbalanced number of patients between the MASH and SS groups
GSE83452	Obese patients undergoing bariatric/diet intervention	28679947	104 MASH/ 44 No MASH	No discrimination possible in the "No MASH" group between healthy and SS patients
GSE59045	Obese patients undergoing bariatric surgery	26028579	5 MASH/ 4 SS	Small group
GSE63067	MAFLD	25993042	8 MASH/ 2 SS	Unbalanced number of patients between the MASH and SS
GSE89632	MAFLD	25581263	19 MASH/ 20 SS	Good MASH and SS patient characterization and balanced number of patients in the 2 groups

Table S2 (A). List of molecules associated with hepatic steatosis and inflammatory response from diseases and functions table obtained in IPA. The molecules in bold were reported as dysregulated genes in MASH versus SS comparison.

Molecule	Disease and Function
**ACOT1**	Hepatic Steatosis
Immunoglobulin	
**SPP1**	
TNF	
TP53	
beta-estradiol	
**ACOT1**	Inflammatory Response
Immunoglobulin	
NOS2	
**SPP1**	
TNF	

Table S2 (B). List of the 58 PPIs obtained by STRING Network analysis.

node1	node2
UBC	TECR
HMGCS2	EHHADH
HIBCH	EHHADH
HMGCS2	PPARA
PPARA	EHHADH
ITIH4	IL2
HNF4A	PPARA
ACOT7	UBC
PRKCE	IL2
ACOT7	TECR
ACOT1	TECR
HMGCS2	UBC
ACOT1	ACOT8
UBC	ACOT8
ACOT1	PRKCE
UBC	CXADR
HIBCH	UBC
UBC	ECH1
HEATR4	ZNF483
ZNF512	HEATR4
ZNF461	HEATR4

HIBCH	ACOT1
ZNF512	ZNF483
UBC	ACOT1
ACOT1	CXADR
HEATR4	ZNF677
UBC	PRKCE
PLSCR2	HEATR4
ZNF512	ZNF677
PLSCR2	ZNF677
PLSCR2	ZNF483
ACOT7	ACOT1
HEATR4	ECH1
ZNF461	ZNF512
ZNF512	ECH1
ZNF483	ECH1
ACOT1	PPARA
HEATR4	ACOT1
PLSCR2	ZNF512
HNF4A	ACOT1
HMGCS2	ACOT1
PPARA	ACOT8
ZNF461	PLSCR2
EHHADH	ACOT8
ACOT1	ITIH4
HMGCS2	ECH1
ACOT1	ECH1
ACOT1	EHHADH
ZNF512	ACOT1
ACOT7	ACOT8
PLSCR2	ACOT1
UBC	ZNF483
ZNF512	UBC
UBC	ZNF677
ZNF677	ACOT1
ZNF483	ACOT1
ACOT1	IL2
ZNF461	ACOT1

Table S3. Betweenness centrality score for PPI network in Figure 1A.

Protein	betweenness score
**ACOT1**	**114.48**
UBC	22.37
ZNF512	2.48
PPARA	1.75
HMGCS2	1.58
ACOT8	1.58
HEATR4	1.53
EHHADH	1.42
ECH1	1.37
PLSCR2	0.7
ZNF483	0.7
PRKCE	0.5
IL2	0.5
ZNF677	0.45
ACOT7	0.33
HIBCH	0.25
ITIH4	0
HNF4A	0
ZNF461	0
TECR	0
CXADR	0

Table S4 (A). List of identified up regulated genes between ACOT1 overexpressing mice and controls, related to metabolism and inflammation. GO = Gene Ontology. PPAR gene targets are in Italics. Genes commonly upregulated in two independent mouse datasets (GSE57290, GSE59042) are underlined.

[redacted]			
**Lipid Metabolism Related Genes**			
**Abcd2**	ATP-binding cassette, sub-family D (ALD), member 2	GO:0006633	fatty acid biosynthetic process
**Abcd2**	ATP-binding cassette, sub-family D (ALD), member 2	GO:0008610	lipid biosynthetic process
**Abcd2**	ATP-binding cassette, sub-family D (ALD), member 2	GO:0006635	fatty acid beta-oxidation
**Abhd2**	abhydrolase domain containing 2	GO:0006634	fatty acid biosynthetic process
**Abhd2**	abhydrolase domain containing 2	GO:0008610	lipid biosynthetic process
**Abhd2**	abhydrolase domain containing 2	GO:0046486	glycerolipid metabolism
**Abhd2**	abhydrolase domain containing 2	GO:0046503	glycerolipid catabolic process
**B3galt1**	UDP-Gal:betaGlcNAc beta 1,3-galactosyltransferase, polypeptide 1	GO:0008610	lipid biosynthetic process
**B3galt1**	UDP-Gal:betaGlcNAc beta 1,3-galactosyltransferase, polypeptide 1	GO:0030148	sphingolipid biosynthetic process
**B3galt1**	UDP-Gal:betaGlcNAc beta 1,3-galactosyltransferase, polypeptide 1	GO:0006665	sphingolipid metabolic process
Cpt1a	*carnitine palmitoyltransferase 1a, liver*	*GO:0006641*	*triglyceride metabolic process*
Cpt1a	*carnitine palmitoyltransferase 1a, liver*	*GO:0006636*	*fatty acid beta-oxidation*
Cpt1a	*carnitine palmitoyltransferase 1a, liver*	*GO:0046486*	*glycerolipid metabolism*
**Dhcr24**	24-dehydrocholesterol reductase	GO:0008610	lipid biosynthetic process
**Fgf21**	fibroblast growth factor 21	GO:0006641	triglyceride metabolic process
**Fgf21**	fibroblast growth factor 21	GO:0046486	glycerolipid metabolism
**Fgf21**	fibroblast growth factor 21	GO:0046505	glycerolipid catabolic process
**Gm12886**	predicted gene 12886	GO:0008610	lipid biosynthetic process
Pck1	*phosphoenolpyruvate carboxykinase 1, cytosolic*	*GO:0006641*	*triglyceride metabolic process*
Pck1	*phosphoenolpyruvate carboxykinase 1, cytosolic*	*GO:0019433*	*triglyceride biosynthetic process*
Pck1	*phosphoenolpyruvate carboxykinase 1, cytosolic*	*GO:0008610*	*lipid biosynthetic process*
Pck1	*phosphoenolpyruvate carboxykinase 1, cytosolic*	*GO:0046486*	*glycerolipid metabolism*
Pck1	*phosphoenolpyruvate carboxykinase 1, cytosolic*	*GO:0045018*	*glycerolipid biosynthetic process*

Pltp	*phospholipid transfer protein*	*GO:0010875*	*positive regulation of cholesterol efflux*
**Sik1**	salt inducible kinase 1	GO:0019434	triglyceride biosynthetic process
**Sik1**	salt inducible kinase 1	GO:0006641	triglyceride metabolic process
**Sik1**	salt inducible kinase 1	GO:0008611	lipid biosynthetic process
**Sik1**	salt inducible kinase 1	GO:0046486	glycerolipid metabolism
**Sik1**	salt inducible kinase 1	GO:0045019	glycerolipid biosynthetic process
**Socs2**	suppressor of cytokine signaling 2	GO:0006650	glycerophospholipid metabolic process
**Socs2**	suppressor of cytokine signaling 2	GO:0046486	glycerolipid metabolism
**Inflammation Related genes**			
**Il1r1**	interleukin 1 receptor, type I	GO:0006954	inflammatory response
**Il1r1**	interleukin 1 receptor, type I	GO:0050727	regulation of inflammatory response
**Abcd2**	ATP-binding cassette, sub-family D (ALD), member 2	GO:1900016	negative regulation of cytokine production involved in inflammatory response
**Cd200**	CD200 antigen	GO:0150079	negative regulation of neuroinflammatory response
**Cd200**	CD200 antigen	GO:0150077	regulation of neuroinflammatory response
**Thbs1**	thrombospondin 1	GO:0006954	inflammatory response
**Lbp**	lipopolysaccharide binding protein	GO:0002232	leukocyte chemotaxis involved in inflammatory response
**Lbp**	lipopolysaccharide binding protein	GO:0060265	positive regulation of respiratory burst involved in inflammatory response
**Epha2**	Eph receptor A2	GO:0006954	inflammatory response

Table S4 (B). IPA associated the dysregulated genes in ACOT1 overexpressing mice with steatosis and identified 5 Top Function-related Networks based on the list of affected genes and their molecular interactors.

Name	p-value range	# Molecules
Liver Steatosis	6.39 e-02 - 4.15e-04	9
Liver Hyperplasia/ Hyperproliferation	1.00e 00 - 4.82e-04	46
Liver Regeneration	1.38 e-03 - 4.15e-04	4
Liver Necrosis/ Cell Death	9.82 e-02 - 7.73 e-03	6
Hepatocellular Carcinoma	1.18e-01 - 1.35e-02	11
**ID**	**Top Diseases and Functions**	**Score**
1	Cancer, Gastrointestinal Disease, Hepatic System Disease	41
2	Cell Death and Survival, Cellular Assembly and Organization, Cellular Compromise	31
3	Cell Signaling, Post-Translational Modification, Protein Synthesis	29
4	Carbohydrate Metabolism, Molecular Transport, Small Molecule Biochemistry	29
5	Cellular Assembly and Organization, DNA Replication, Recombination, and Repair, Cell Death and Survival	29
6	Connective Tissue Development and Function, Organ Morphology, Organismal Development	27
7	Cell Cycle, Cell Morphology, Cellular Assembly and Organization	20
8	Small Molecule Biochemistry, Amino Acid Metabolism, Carbohydrate Metabolism	10

Table S5(A). List of the DEGs identified between ACOT1 overexpressed mice and the control group.

SYMBOL	logFC	FC	P.Value
Olfr698	-1.29	0.41	1.81E-06
Gm3336	-0.75	0.60	3.90E-05
Cox6b2	-0.53	0.69	0.000163585
Gm9731	-0.53	0.69	0.000207883
1700007K13Rik	-0.91	0.53	0.000262946
Shisa8	-0.77	0.59	0.000265583
Gm20257	-0.72	0.61	0.000434453
Mt2	1.87	3.66	0.000467494
Gm13657	-0.90	0.54	0.000473701
Obp2a	-3.13	0.11	0.000502065
Itih3	0.64	1.56	0.000733369
Gm4841	-0.91	0.53	0.000748197
Tmed2	-0.60	0.66	0.000753493
Fga	0.65	1.57	0.000804964
Gm12886	0.77	1.70	0.000859515
Fkbp11	-0.78	0.58	0.000888804
9230114K14Rik	-0.87	0.55	0.000905232
Dkk4	-1.12	0.46	0.000931385
Sdr9c7	-0.76	0.59	0.00108376
Mir365-1	-0.66	0.63	0.0013036
Olfr498	-0.70	0.62	0.00137098
Gm10030	-0.65	0.64	0.00143284
Hist1h1c	-0.69	0.62	0.00161319
Zfp992	-1.34	0.40	0.00166242
Osmr	0.65	1.57	0.00170123
Slc37a1	0.59	1.50	0.00170211
Dpy30	-0.53	0.69	0.00174489
Mir3098	-0.73	0.60	0.00184681
Lrg1	0.75	1.69	0.00202303
Gstp1	-0.57	0.67	0.00221514
Gstp3	-0.86	0.55	0.00232101
Zfp982	-1.18	0.44	0.00233088
Hsd3b2	-0.52	0.70	0.00233881
Mt1	1.17	2.24	0.00234968
Qsox1	0.65	1.57	0.00235972

Itih4	0.75	1.68	0.0024251
Cpt1a	0.78	1.72	0.00249999
Gria3	-0.87	0.55	0.00254027
Cyp4b1-ps2	-0.74	0.60	0.00275429
Hist1h3c	-1.16	0.45	0.00281231
Hist1h4h	-0.77	0.59	0.00284321
Plce1	0.63	1.55	0.00290387
Phlda1	-0.93	0.53	0.00314733
Ass1	0.65	1.57	0.00324039
Ddias	-1.14	0.45	0.00332312
Snord58b	-0.92	0.53	0.00341007
Ndufb3	-0.67	0.63	0.00365103
Steap4	0.73	1.66	0.00366607
Hist1h4i	-1.51	0.35	0.00388738
Rpl27a	-0.73	0.60	0.0040275
Csprs	-0.88	0.54	0.00449131
Hspb11	-0.67	0.63	0.00460633
Fgl1	0.69	1.62	0.00464524
Neat1	0.75	1.69	0.00486417
Keg1	-0.70	0.62	0.00492597
Sik1	0.59	1.50	0.00496611
Gm13021	-0.86	0.55	0.00497166
Olfr521	-0.56	0.68	0.00544099
Saa3	-1.57	0.34	0.00546101
Oaz1	-0.68	0.63	0.00558033
Oog3	-0.74	0.60	0.00562788
Coa3	-0.53	0.69	0.00562934
Olfr373	-0.88	0.54	0.00577996
Snord70	-0.54	0.69	0.00598362
Gm8465	-0.60	0.66	0.00598876
Zfp991	-1.25	0.42	0.00602595
Atp8b5	-0.55	0.68	0.00608947
Epha2	0.82	1.76	0.00620264
Blvrb	-0.56	0.68	0.00629849
Smcp	-1.14	0.46	0.00641265
Vmn2r109	-0.52	0.70	0.00666864
Serpina3n	0.73	1.66	0.00669675
Zfp979	-1.32	0.40	0.00685155
Chchd1	-0.75	0.60	0.00728604

Ccl6	-0.57	0.67	0.00735202
Rps27l	-0.69	0.62	0.00743155
Tacc2	0.64	1.56	0.00764904
S100g	-0.70	0.62	0.00778066
Cfap53	-0.60	0.66	0.00800126
Olfr299	-0.82	0.56	0.00814587
5730507C01Rik	-0.77	0.59	0.00827728
2810474O19Rik	-0.86	0.55	0.00859063
Vmn2r122	-0.71	0.61	0.00860009
Tmem141	-0.52	0.70	0.00860845
Snhg15	-0.68	0.62	0.00881302
Snord52	-0.77	0.59	0.00893357
0610040B10Rik	-0.65	0.64	0.00912353
Tyw5	-0.53	0.69	0.00914264
Zfp874a	-0.60	0.66	0.00946609
Vmn1r238	-0.52	0.70	0.00961465
Endog	-0.55	0.68	0.00984307
Nat8	-0.52	0.70	0.00988086
Abracl	-0.68	0.63	0.00996595
Hist1h2ad	-0.73	0.60	0.0101892
Dmac1	-0.59	0.66	0.0103781
Rps19-ps3	-0.75	0.60	0.0106466
Zbtb16	0.71	1.64	0.0109764
Igfbp1	1.02	2.03	0.0112638
Igk	1.48	2.83	0.0113105
Apol7b	-0.53	0.69	0.0116889
Nipal1	0.83	1.78	0.0116959
1810046K07Rik	-0.66	0.63	0.011705
Il1r1	0.93	1.91	0.0121411
Sult2a7	-0.78	0.58	0.0121824
Snord8	-0.67	0.63	0.0121915
Hist1h1d	-0.95	0.52	0.0123409
Gm10033	-0.63	0.65	0.012543
Saa2	1.37	2.58	0.0125687
Emg1	-0.57	0.67	0.0125889
4930529C04Rik	-0.65	0.64	0.0127211
Gpx3	0.78	1.71	0.0129019
Mup6	-0.59	0.67	0.0129029
Eda2r	-0.85	0.56	0.0131168

Mir122	1.08	2.12	0.0131732
Chil4	-0.53	0.69	0.0132794
Prg4	0.89	1.85	0.0133066
Olfr657	-0.68	0.63	0.0133124
Lbp	0.77	1.71	0.0133233
Ighm	0.69	1.62	0.0134035
Pfdn5	-0.58	0.67	0.0135895
Gm5617	-0.58	0.67	0.0138777
Med31	-0.61	0.66	0.0139053
Gbp2b	-0.70	0.62	0.0142348
Hist1h3b	-0.56	0.68	0.0145062
Gm19705	-0.69	0.62	0.0145142
9430065F17Rik	-0.54	0.69	0.0146532
Sec11c	-0.55	0.68	0.0146921
2010107E04Rik	-0.58	0.67	0.0149936
Mfsd2a	1.04	2.06	0.0150371
Cd200	0.69	1.62	0.0150589
Gm3255	-0.61	0.65	0.0151383
Snord16a	-1.20	0.43	0.0153357
Snrpe	-0.59	0.66	0.0154141
9030619P08Rik	-1.04	0.48	0.0154323
Slc16a5	0.62	1.53	0.0160255
LOC105246765	-0.54	0.69	0.0160589
Vamp7	-0.54	0.69	0.0162255
Gm14327	-0.62	0.65	0.016244
Thbs1	0.63	1.54	0.016399
Lcn2	1.43	2.70	0.0165405
Glod5	-1.09	0.47	0.0167419
Hist1h2bh	-0.68	0.62	0.016783
Sucnr1	-0.59	0.67	0.0170907
Snrpg	-0.56	0.68	0.0175269
Igkv4-68	0.70	1.62	0.0176185
Smim26	-0.54	0.69	0.0176326
Cdkn1a	-1.15	0.45	0.018176
Tmem267	0.65	1.57	0.0182348
Hist1h3d	-0.56	0.68	0.0184481
Olfr877	-0.58	0.67	0.0188213
Fam96a	-0.54	0.69	0.0192667
Aym1	0.62	1.53	0.0193399

Mir3475	-0.65	0.64	0.0194472
Plin4	0.97	1.96	0.0195192
Usp17la	-0.54	0.69	0.0196861
Ighg	1.61	3.06	0.0196957
Znhit3	-0.53	0.69	0.0200336
Mir200c	-0.53	0.69	0.0200681
Tmem126a	-0.52	0.70	0.0201981
Nudcd2	-0.54	0.69	0.0202633
Slc3a1	0.64	1.55	0.0203846
Rps13	-0.72	0.61	0.0204995
5033403H07Rik	-0.80	0.57	0.0205027
Capn8	-0.86	0.55	0.0206173
Sncg	0.73	1.66	0.0206771
Nudt7	-0.63	0.64	0.0207919
Mup-ps12	-0.74	0.60	0.0215988
Gm14147	-0.73	0.60	0.0218621
Serpina6	0.72	1.64	0.021918
Mir3070b	-0.61	0.66	0.0219318
Gm9112	-0.79	0.58	0.0220023
Gm11695	0.94	1.92	0.0226105
Abhd2	0.82	1.77	0.0232808
Iglv1	0.71	1.64	0.0233218
Igh-V3609N	0.61	1.52	0.023555
Skor2	-0.52	0.70	0.0242325
Olfr1141	-0.59	0.67	0.0245422
Hist4h4	-0.55	0.68	0.0247367
Foxq1	-0.98	0.51	0.0247511
Cks2	-0.60	0.66	0.0248133
Rec8	-0.69	0.62	0.0254775
Gas6	0.64	1.56	0.0258933
Snord1b	-0.75	0.59	0.0259249
Snord69	-0.77	0.59	0.0259737
Zfp600	-0.66	0.63	0.0263421
Mup5	-0.53	0.69	0.0267346
Zfas1	-0.69	0.62	0.0272264
Igh-VJ558	0.91	1.95	0.0276924
Mrpl53	-0.68	0.62	0.027802
I830127L07Rik	-0.53	0.69	0.0283046
2310001H17Rik	-0.54	0.69	0.0284939

Rpp40	-1.29	0.41	0.0288954
Gm4758	-0.60	0.66	0.0289073
Omd	-0.62	0.65	0.0289303
Pck1	0.70	1.63	0.0291105
Orm2	1.33	2.51	0.029611
Fgf21	0.64	1.56	0.0297674
Slc6a8	0.62	1.54	0.029818
Snora15	-1.46	0.36	0.0305305
Snord85	-0.72	0.61	0.0308796
Art2b	-0.58	0.67	0.0319546
A630038E17Rik	-0.68	0.62	0.0325736
Olfr116	-0.53	0.69	0.033253
Dnajc15	-0.63	0.65	0.0333318
Olfr986	-0.55	0.68	0.0337956
Scarna9	-0.63	0.64	0.0341911
Olfr1316	-0.61	0.65	0.0348176
Itgb1bp2	0.59	1.50	0.0354705
Mup21	-1.06	0.48	0.0356552
Dhcr24	0.60	1.52	0.0360531
Hist1h2bg	-0.74	0.60	0.0363228
Snord22	-1.21	0.43	0.0363295
Igkv3-4	0.73	1.65	0.0363482
Chac2	-0.61	0.65	0.0369618
Rdh18-ps	0.59	1.50	0.0370981
9430037G07Rik	-0.59	0.66	0.0371906
C7	0.81	1.76	0.038173
Trp53inp1	-0.59	0.66	0.0387651
B3galt1	0.76	1.69	0.0390581
Rps25	-0.62	0.65	0.0394716
Lyve1	0.65	1.57	0.040345
Ccng1	-0.63	0.65	0.0407956
Ifi27	-0.66	0.63	0.0415999
Mirlet7f-1	0.97	1.96	0.0423372
Selenbp2	-0.92	0.53	0.0423683
Snord19	-0.86	0.55	0.0425297
Etaa1os	-0.64	0.64	0.0426104
Rps15a-ps5	-0.54	0.69	0.0429092
Abcd2	0.99	1.98	0.0431446
Gm6445	-0.81	0.57	0.0433024

Aplp2	0.78	1.72	0.0436742
Socs2	1.00	2.00	0.0439303
Try10	-0.65	0.64	0.0440709
Sult1b1	-0.54	0.69	0.0471675
Tcrg-V4	-0.53	0.69	0.0473729
Hist1h2ai	-0.52	0.70	0.0474312
Zfp108	-0.56	0.68	0.0478461
Ighv1-19	1.31	2.48	0.0485722
1700080G11Rik	-0.57	0.68	0.0495055

Table S5(B). List of the DEGs identified between ACOT1 silenced mice and the control group.

SYMBOL	logFC	FC	P.Value
Zbtb16	-1.52	0.35	0.00001
Acot1	-1.05	0.48	0.00001
gene	0.97	1.96	0.00002
Sytl5	-1.16	0.45	0.00002
Cyp7a1	1.62	3.07	0.00003
LOC105245453/Gm2399	-1.41	0.38	0.00003
Cyp2c50	1.41	2.66	0.00004
C230037L18Rik	-0.90	0.54	0.00004
Traj58	1.04	2.06	0.00006
Selenbp2	1.77	3.41	0.00006
Mfsd2a	-0.98	0.51	0.00007
Nid1	-1.17	0.44	0.00008
Anxa2	-1.36	0.39	0.00009
Sult2a8	0.96	1.95	0.00009
Mthfd1l	-1.00	0.50	0.00009
Ces3b	1.22	2.33	0.00012
Gm10319	0.80	1.74	0.00014
Gas6	-0.93	0.52	0.00014
Slc22a7	2.01	4.03	0.00015
2010003K11Rik	-1.50	0.35	0.00015
Slc35g1	-0.82	0.57	0.00015
AI987944	0.79	1.73	0.00017
Dusp3	-0.81	0.57	0.00018
Bhmt-ps1	1.12	2.18	0.00018
Acot3	-1.55	0.34	0.00019
Cbr1	-1.48	0.36	0.00019
Sult5a1	0.91	1.89	0.00019
Alpl	-0.69	0.62	0.00021
Plin5	-0.67	0.63	0.00022
Rragd	-0.70	0.61	0.00023
Hsd3b5	4.35	20.40	0.00023
Iqgap1	-0.72	0.61	0.00023
Timp3	-0.75	0.60	0.00024
Mup9	0.97	1.97	0.00026
Gm10680	-0.79	0.58	0.00027

Cbr1	-1.27	0.42	0.00027
Lrrc39	-1.12	0.46	0.00027
Col1a1	-0.89	0.54	0.00029
Myo1d	-0.67	0.63	0.00029
Pdk4	-1.34	0.40	0.00029
Hsd3b4/Gm10681	2.34	5.07	0.00030
Hsd3b4/Gm10681	2.34	5.07	0.00030
Cntnap1	-0.90	0.53	0.00030
Gpnmb	-1.24	0.42	0.00032
Sox5it	-0.78	0.58	0.00034
Mup21	1.30	2.46	0.00034
Adh6-ps1	1.27	2.42	0.00037
Sprr1a	-2.27	0.21	0.00039
Gpcpd1	1.10	2.14	0.00039
Id1	-0.93	0.52	0.00039
Traj6	0.76	1.69	0.00040
Gm13522	0.89	1.85	0.00040
Unc119	-0.73	0.60	0.00041
Capn8	1.15	2.23	0.00047
Cxcl16	-0.63	0.65	0.00049
Ipw	0.85	1.81	0.00049
Cyp39a1	-1.03	0.49	0.00049
Gnat1	0.90	1.87	0.00050
C3ar1	-0.63	0.64	0.00050
Tinag	-1.16	0.45	0.00052
Gm14296/2210418O10Rik	0.76	1.69	0.00052
Slc43a1	1.34	2.53	0.00054
Prok1	0.66	1.58	0.00054
Gm25083	0.98	1.97	0.00055
Scd2/Mir5114	-1.44	0.37	0.00058
Renbp	-0.60	0.66	0.00061
F11	0.78	1.72	0.00062
Gprc5b	-1.41	0.38	0.00062
Nnmt	1.12	2.17	0.00062
Crim1	-0.70	0.61	0.00064
Gstm3	-1.79	0.29	0.00064
Rbm12b1/Rbm12b2	0.68	1.61	0.00065
Aqp7	-0.81	0.57	0.00066
Cyp7b1	1.37	2.58	0.00068

Cyp4a12b	1.00	2.00	0.00069
Fmo2	-0.88	0.54	0.00070
Tmem86b	0.75	1.68	0.00070
Fgl1	1.27	2.41	0.00071
Hspb1	-0.58	0.67	0.00073
Ttc39a	-0.70	0.62	0.00074
Gm22768	0.61	1.53	0.00074
Mup11/Mup10	0.84	1.79	0.00075
Spink1	-0.79	0.58	0.00075
Gm23168	1.33	2.52	0.00075
Sox4	-0.86	0.55	0.00078
Cyp2c54	2.14	4.41	0.00080
Tbc1d8	-0.63	0.64	0.00081
Pex11a	-0.91	0.53	0.00082
App	-0.71	0.61	0.00082
Gm11695	-1.27	0.42	0.00084
Smim8	0.64	1.56	0.00084
Plk3	-0.85	0.56	0.00085
Gm13773	0.66	1.58	0.00088
3110082I17Rik	0.79	1.73	0.00088
Tnfrsf12a	-0.80	0.58	0.00090
Olfr845	0.88	1.85	0.00091
Sparc	-0.59	0.67	0.00092
Tax1bp3	-0.55	0.68	0.00092
Cyp2c38	-1.09	0.47	0.00093
Abcb1a	-1.05	0.48	0.00093
Zfp960	0.80	1.75	0.00093
Abcd2	-1.14	0.45	0.00094
Sult1b1	0.87	1.83	0.00095
Gm26067	-0.80	0.57	0.00096
Tmem86a	-0.78	0.58	0.00096
Gm15754	0.66	1.58	0.00099
Olfr1487	-0.66	0.63	0.00100
Spon2	-0.71	0.61	0.00100
Slco1a4	-0.95	0.52	0.00100
Mmp12	-1.34	0.40	0.00101
Crat	-0.87	0.55	0.00103
Hist1h4c	0.81	1.76	0.00104
Vat1	-0.60	0.66	0.00106

Neb	0.71	1.64	0.00106
B3galt1	0.67	1.59	0.00109
n-R5s64	0.72	1.65	0.00109
Gm23326	0.74	1.67	0.00111
Platr16	0.99	1.99	0.00112
Itih3	0.77	1.71	0.00114
Cyp2d9	0.91	1.88	0.00114
Gsta4	-1.02	0.49	0.00115
Myo1c	-0.59	0.66	0.00118
Snora64/Rps2	0.82	1.76	0.00119
Lpin2	-0.91	0.53	0.00120
Piezo1	-0.63	0.65	0.00120
Mtmr11	-0.81	0.57	0.00120
Lifr	0.77	1.70	0.00120
Rcan1	-0.76	0.59	0.00121
Foxl1	0.68	1.61	0.00126
Gm24788	0.69	1.61	0.00126
Ppl	-0.59	0.66	0.00126
Gm24843	0.90	1.87	0.00127
Cidec	-2.33	0.20	0.00129
Cpt1b/Chkb/ChkbCpt1b	-0.86	0.55	0.00129
Gbp11	1.00	2.00	0.00130
Galk1	-0.59	0.67	0.00130
Anxa3	-0.63	0.64	0.00130
Flnb	-0.56	0.68	0.00136
Map4k4	-0.62	0.65	0.00136
Pak1	-0.62	0.65	0.00137
Mpv17l	0.62	1.54	0.00138
Gm25260	-0.89	0.54	0.00142
Irf2bp2	-0.62	0.65	0.00144
Ttc39c	0.85	1.80	0.00145
Lrtm1	1.09	2.12	0.00145
C1ql4	-0.96	0.51	0.00145
Gm23503	0.68	1.60	0.00145
Mmp14	-0.56	0.68	0.00146
Pik3ap1	-0.54	0.69	0.00146
Plin4	-1.12	0.46	0.00147
Got1	0.72	1.65	0.00148
Mgst3	-1.11	0.46	0.00150

Gm25008	1.00	2.00	0.00150
Hsd3b3	0.67	1.59	0.00151
Slc51b	-0.98	0.51	0.00151
Osmr	-0.53	0.69	0.00152
Prss8	-0.77	0.59	0.00153
Ephb2	-0.73	0.60	0.00153
Mfge8	-0.61	0.65	0.00156
Limk1	-0.68	0.63	0.00156
Oat	0.65	1.57	0.00159
Hsd17b2	0.60	1.51	0.00163
Sult1e1	2.05	4.14	0.00164
Sdcbp2	-0.63	0.65	0.00164
Tlr13	-0.67	0.63	0.00165
Nrep	0.74	1.67	0.00168
1810046K07Rik	1.10	2.15	0.00170
Arsa	-0.59	0.67	0.00171
Cers6	-0.99	0.50	0.00172
Trim30d	0.75	1.68	0.00173
Gm23099	-0.77	0.59	0.00177
Cyp4a12a	0.59	1.50	0.00178
Abcc4	-0.62	0.65	0.00180
Fam83c	0.62	1.53	0.00180
Ttc39aos1	-0.78	0.58	0.00182
Gm9992	-0.59	0.67	0.00184
Pea15a	-0.52	0.70	0.00184
St3gal5	-0.57	0.67	0.00189
Gm14296/2210418O10Rik	0.69	1.62	0.00190
Rhpn2	-0.71	0.61	0.00195
Slc12a4	-0.54	0.69	0.00197
Gm12676/LOC102637577	0.77	1.70	0.00197
Gm24762	-0.94	0.52	0.00201
Mup12	0.64	1.55	0.00203
Gga2	-0.56	0.68	0.00205
Prtn3	0.75	1.68	0.00206
n-R5s168	0.74	1.67	0.00207
n-R5s166	0.67	1.59	0.00207
Leprot	-0.67	0.63	0.00208
Cd14	-0.63	0.65	0.00209
Serpina1e	1.53	2.89	0.00210

Inca1	0.71	1.64	0.00211
Ly6d	-1.93	0.26	0.00212
Traj18	-0.52	0.70	0.00213
Clcn6	-0.65	0.64	0.00214
Emp2	-0.59	0.66	0.00218
Pcdh17	-0.73	0.60	0.00218
Gstm1	-0.72	0.61	0.00220
Nrp1	0.72	1.64	0.00232
Snora44	1.51	2.85	0.00235
Ghrl	0.76	1.69	0.00235
Col6a3	-0.65	0.64	0.00239
Gm26608	0.97	1.96	0.00240
Sptan1	-0.58	0.67	0.00242
Cd93	-0.54	0.69	0.00252
Agxt	0.67	1.59	0.00260
BC089597	0.61	1.53	0.00262
Cd9	-1.04	0.49	0.00266
Gm14412	0.86	1.81	0.00266
Kctd12	-0.56	0.68	0.00267
Tead1	-0.58	0.67	0.00271
Tmc7	-0.70	0.61	0.00275
Slc16a7	-0.81	0.57	0.00276
Col3a1	-0.80	0.57	0.00276
Ubqln4	-0.55	0.68	0.00278
3110043O21Rik	-0.61	0.65	0.00279
Fam163b	0.71	1.63	0.00280
Tmem98	-0.52	0.70	0.00282
Nrg1	-1.16	0.45	0.00285
Gadd45b	-0.65	0.64	0.00285
Zgrf1	0.75	1.69	0.00285
Acot2	-2.51	0.18	0.00287
Gm23407	-0.61	0.66	0.00291
Gm6444	0.59	1.51	0.00292
Pdcd5	-0.53	0.69	0.00295
Gm14816	0.64	1.56	0.00298
Gm25401	0.66	1.57	0.00300
Slc22a26	-0.68	0.62	0.00301
E330011O21Rik	-0.62	0.65	0.00302
Gadd45g	-0.75	0.59	0.00304

Gm11467	0.62	1.53	0.00304
Gm23534	0.97	1.96	0.00304
Olfr370	0.71	1.64	0.00304
Mybl1	-1.13	0.46	0.00304
Cd3g	-0.76	0.59	0.00306
C1s1	0.61	1.53	0.00318
Pla2g6	-0.70	0.61	0.00322
n-R5s152	0.73	1.66	0.00322
Rps20	1.11	2.15	0.00322
Gm23136	0.71	1.64	0.00324
Lonrf3	-0.61	0.65	0.00325
Trav6-1	0.77	1.70	0.00329
Prss23os	-0.64	0.64	0.00335
Mgll	-0.61	0.66	0.00339
Lyve1	-0.62	0.65	0.00339
Vldlr	-0.71	0.61	0.00340
Hk2	-0.64	0.64	0.00343
Nnt	-0.54	0.69	0.00343
C8b	0.93	1.91	0.00344
Gm13680	0.73	1.65	0.00346
Aadat	0.75	1.68	0.00348
Prrg4	-0.65	0.64	0.00349
Elovl7	-0.70	0.61	0.00351
Anxa7	-0.52	0.70	0.00352
Gm16063	0.67	1.59	0.00352
Cd63	-0.70	0.62	0.00353
Rps13-ps2/Rps13	0.93	1.91	0.00353
Map3k13	-0.52	0.70	0.00353
Btg2	-0.78	0.58	0.00355
H2-Q1	-0.87	0.55	0.00356
n-R5s85	0.99	1.99	0.00360
Osbpl3	-1.19	0.44	0.00365
Ube2u	0.85	1.80	0.00365
Vim	-0.60	0.66	0.00368
Ccdc120	-0.55	0.69	0.00370
Igkv6-25	-2.31	0.20	0.00376
Trbv17	0.66	1.58	0.00377
Mir667	0.95	1.93	0.00378
Lrp4	-0.56	0.68	0.00379

Kidins220	-0.57	0.67	0.00381
Gm23730	0.88	1.84	0.00385
Cgref1	-0.83	0.56	0.00386
Mvp	-0.68	0.62	0.00388
Hspa1a/Hspa1b	-0.86	0.55	0.00389
Slc16a5	-0.74	0.60	0.00390
Lamc1	-0.52	0.70	0.00392
Haus8	-0.80	0.57	0.00393
Slc5a6	-0.56	0.68	0.00404
Olfr1205	0.69	1.62	0.00412
Slc6a9	-0.55	0.68	0.00413
Gm16794	0.59	1.50	0.00431
Rhoj	-0.51	0.70	0.00443
Emp1	-0.70	0.62	0.00443
Gm23182	0.63	1.55	0.00444
Gpc6	-0.58	0.67	0.00444
Rhoc	-0.52	0.70	0.00449
Ehd2	-0.55	0.69	0.00456
Gm22155	1.08	2.11	0.00457
Srgap2	-0.53	0.69	0.00465
Rhox2-ps	0.64	1.56	0.00472
Susd4	1.52	2.88	0.00474
Wbp5	-0.57	0.67	0.00478
Dusp6	-0.61	0.66	0.00481
Stra6	0.68	1.60	0.00482
Acot4	-1.12	0.46	0.00488
Rmnd1	-0.56	0.68	0.00490
Vmn1r103	0.98	1.97	0.00492
Slc22a28	0.82	1.76	0.00494
Mafk	-0.59	0.67	0.00499
Rec114	0.60	1.52	0.00507
Tmem45b	-0.63	0.64	0.00508
Sult1d1	0.70	1.62	0.00508
Tm4sf4	-0.81	0.57	0.00508
Jun	-1.13	0.46	0.00509
Gm23422	0.71	1.64	0.00511
Muc1	-0.54	0.69	0.00511
Cyp2d41-ps	0.67	1.59	0.00515
3110045C21Rik	0.61	1.53	0.00526

Saa1	1.93	3.82	0.00526
Abhd2	-0.79	0.58	0.00527
Pgm1	-0.53	0.69	0.00532
Gm10447	0.62	1.54	0.00532
Gm17229/4930403O15Rik	0.63	1.54	0.00536
A630089N07Rik	0.63	1.55	0.00539
Eif2ak3	-0.55	0.68	0.00539
Gm6665	-0.73	0.60	0.00542
Pltp	-0.87	0.55	0.00546
Cygb	-0.56	0.68	0.00553
AB010352	0.86	1.82	0.00560
Vcam1	-0.59	0.66	0.00564
Plet1	-0.59	0.67	0.00565
Psd4	-0.53	0.69	0.00565
Rnf24	-0.56	0.68	0.00568
Fgf21	-0.91	0.53	0.00568
Mir466n	1.08	2.11	0.00570
Scd3	-0.60	0.66	0.00571
Hes1	-0.52	0.70	0.00576
Defb1	-0.77	0.59	0.00582
Gm4450	0.78	1.72	0.00584
Hist1h2ab	-0.80	0.57	0.00588
Gm22673	0.65	1.57	0.00589
Gm10718	1.01	2.01	0.00596
Lncbate1	0.78	1.72	0.00600
Klf6	-0.96	0.51	0.00604
Zwilch	-0.55	0.68	0.00606
Gm5292	0.59	1.50	0.00612
Col1a2	-0.53	0.69	0.00613
Gm5327	0.72	1.65	0.00614
Morc4	-0.55	0.68	0.00617
Hist1h2bf	0.67	1.59	0.00617
Gnmt	0.70	1.62	0.00622
Dsg1a	0.87	1.83	0.00623
Efnb2	-0.54	0.69	0.00623
Kdsr	-0.57	0.68	0.00624
Idi1	0.59	1.50	0.00625
Olfr412	0.84	1.78	0.00627
Cyp1a2	0.61	1.52	0.00628

Lect1	0.61	1.53	0.00636
Gm23984	0.78	1.71	0.00636
Lbh	-0.52	0.70	0.00637
Rarres1	0.75	1.69	0.00650
Cd34	-0.56	0.68	0.00650
Gm6669	0.86	1.82	0.00654
Nr1i2	-0.53	0.69	0.00661
Snord99	0.79	1.73	0.00662
BC024386	0.59	1.50	0.00663
Grap	-0.56	0.68	0.00668
Mir297a-2	0.62	1.54	0.00672
Rpl5	0.99	1.99	0.00672
Mmp13	-0.84	0.56	0.00673
Col6a6	0.60	1.51	0.00674
Gja1	-0.52	0.70	0.00674
5830417I10Rik	0.68	1.60	0.00676
Cd4	0.78	1.72	0.00678
Agpat9	-0.78	0.58	0.00681
Msmo1	0.64	1.56	0.00684
Plin2	-0.63	0.65	0.00688
Bsn	0.59	1.50	0.00689
Lilrb4a	-0.58	0.67	0.00689
Mir1291/Snora34	0.72	1.64	0.00695
Mir34a	0.80	1.74	0.00696
Gm13556	0.73	1.66	0.00700
Gm3543	-0.62	0.65	0.00703
Lepr	-0.67	0.63	0.00711
Ets2	-0.52	0.70	0.00712
Epha7	-0.54	0.69	0.00713
Fzd8	0.63	1.55	0.00725
Mup-ps12	0.64	1.56	0.00734
Gm6900	0.87	1.83	0.00746
Havcr2	-0.66	0.63	0.00753
Dsp	-0.52	0.70	0.00759
Gm22681	-0.55	0.68	0.00761
Slc22a5	-0.53	0.69	0.00762
Snord70	1.09	2.12	0.00765
Gm23822	0.59	1.51	0.00774
Scnn1a	0.71	1.63	0.00784

Tbx20	-0.55	0.68	0.00790
Gm25039	0.93	1.90	0.00793
Serpine2	0.63	1.55	0.00815
Spp1	-0.61	0.66	0.00816
Gm10715	0.61	1.53	0.00817
Igfals	0.59	1.50	0.00821
Ermp1	-0.64	0.64	0.00825
Ccl9	-0.52	0.70	0.00826
Snord68/Rpl13	1.28	2.42	0.00830
Gm6139	0.74	1.67	0.00834
Rgs4	-0.54	0.69	0.00835
Gm8363	0.64	1.56	0.00836
Htatip2	-0.59	0.66	0.00838
Anxa5	-0.77	0.59	0.00839
Otud1	-0.66	0.63	0.00846
H2-M2	-0.65	0.64	0.00853
08-Mar	-0.57	0.68	0.00880
Comtd1	-0.75	0.59	0.00889
Chrnb1	-0.72	0.61	0.00890
Tkt/Mir3076	-0.53	0.69	0.00893
Ksr1	-0.52	0.70	0.00893
Klk1b4	-0.76	0.59	0.00893
Fam83a	-0.90	0.53	0.00894
Wfdc21	0.70	1.62	0.00908
Cbr3	-0.92	0.53	0.00910
Igkv4-56	0.63	1.55	0.00925
Egfr	1.05	2.07	0.00942
Sdf2l1	0.71	1.64	0.00949
Nek2	-0.53	0.69	0.00949
Grm4	0.60	1.51	0.00950
Cln6	-0.61	0.65	0.00954
Gsta1/Gm3776	-1.61	0.33	0.00958
Enc1	-0.74	0.60	0.00959
Slc22a27	-1.89	0.27	0.00962
Gm24276	0.89	1.85	0.00962
Epb41l1	-0.52	0.70	0.00971
Hsd17b6	-1.33	0.40	0.00984
Gm15622	0.63	1.55	0.00985
Gm4987	0.82	1.76	0.00994

Dhrs9	-0.55	0.68	0.01001
Casc4	-0.58	0.67	0.01002
Fam25c	0.75	1.68	0.01003
Plekha1	-0.58	0.67	0.01004
A130010J15Rik	-0.64	0.64	0.01012
Top2a	-0.56	0.68	0.01015
n-R5s127	1.03	2.05	0.01018
Pyroxd2	-0.56	0.68	0.01022
Hspa2	-0.53	0.69	0.01023
Gm22127	0.70	1.63	0.01030
Gm25606	0.63	1.55	0.01032
Jund	-0.62	0.65	0.01047
Lipg	-0.62	0.65	0.01051
Igfbp1	-1.19	0.44	0.01056
9130401M01Rik	-0.52	0.70	0.01060
Plekhb2	-0.53	0.69	0.01061
Hist1h4d	0.92	1.89	0.01064
Ccdc138	0.61	1.52	0.01065
Sorbs3	0.60	1.51	0.01069
Gm23587	0.62	1.54	0.01072
Nt5e	-0.75	0.60	0.01082
Gm23264	0.60	1.52	0.01087
4930405O22Rik	-0.55	0.68	0.01089
Gm25776	-0.98	0.51	0.01107
Gm26489	1.03	2.05	0.01116
B4galt5	-0.69	0.62	0.01118
Pdgfra	-0.58	0.67	0.01120
Cmtm4	-0.58	0.67	0.01126
Gpx6	1.05	2.08	0.01128
Olfr373	0.76	1.69	0.01132
Gm26491	-0.92	0.53	0.01135
Orm2	1.18	2.27	0.01139
Gm15723	0.65	1.57	0.01145
Adgrg2	-0.88	0.54	0.01157
LOC102642071	0.64	1.56	0.01163
Gsta1/Gm3776	-0.86	0.55	0.01169
Tmem184c	0.62	1.54	0.01171
Ccnd1	-0.72	0.61	0.01172
Nt5c2	-0.56	0.68	0.01176

Rps8/Snord38a	0.74	1.68	0.01176
Krt23	-1.00	0.50	0.01177
D17H6S56E-5	-0.53	0.69	0.01181
Gm25514	0.61	1.52	0.01183
Gm5523	0.62	1.53	0.01190
Gm22368/Gm8814	0.71	1.64	0.01198
Aox1	-0.77	0.59	0.01199
Gm23008	0.72	1.65	0.01203
Serpinb6b	-0.57	0.68	0.01208
Gm24253	0.99	1.98	0.01210
Gm14601/Gm15482	0.68	1.60	0.01213
Frzb	-0.53	0.69	0.01233
Gm16277	0.61	1.53	0.01246
Gm24588	0.67	1.60	0.01249
Ugcg	-0.55	0.68	0.01269
Anxa1	-0.60	0.66	0.01272
Vmn2r41	0.83	1.77	0.01289
Igfbp5	-0.86	0.55	0.01297
Cadm4	0.63	1.55	0.01298
Gm26905	0.63	1.54	0.01315
Olfr765	0.87	1.83	0.01318
Gm4224	0.87	1.82	0.01318
Gm24780	1.01	2.01	0.01325
Crybb3	0.61	1.53	0.01326
Cyp2c37	1.24	2.37	0.01330
Arhgap11a	-0.70	0.61	0.01332
Lrp2bp	-0.57	0.67	0.01359
Sbds	-0.53	0.69	0.01360
Slc16a1	-0.63	0.65	0.01372
Serpine1	-0.74	0.60	0.01374
Tmc5	0.64	1.55	0.01378
Gimap5	-0.52	0.70	0.01379
Saa2	2.04	4.12	0.01380
Fkbp14/Gm38818	-0.62	0.65	0.01389
Slc16a13	-0.63	0.65	0.01394
Gm23546	1.00	2.00	0.01404
Trav9-2	0.70	1.63	0.01408
Unc119b	-0.57	0.68	0.01423
Cyp17a1	-0.54	0.69	0.01438

LOC102641711	0.72	1.65	0.01460
Mir328	0.59	1.50	0.01469
Rgs5	-0.52	0.70	0.01475
Gm5093	0.66	1.58	0.01477
Snord1c	0.91	1.88	0.01485
Mageh1	-0.58	0.67	0.01485
Gm8203	0.60	1.51	0.01487
Gm10268	0.70	1.62	0.01506
Gm14405	0.75	1.69	0.01515
Aqp4	-0.57	0.68	0.01517
Gm10639	-0.94	0.52	0.01518
Snord83b	0.80	1.74	0.01540
Trappc6a	-0.56	0.68	0.01542
Ms4a4a	-0.60	0.66	0.01545
S100a6	-0.54	0.69	0.01550
1700080G11Rik	0.71	1.63	0.01561
Lrg1	0.69	1.61	0.01561
Gclc	-0.54	0.69	0.01568
Gm19619	-0.73	0.60	0.01569
Gm17361	0.72	1.65	0.01572
Ifit1bl1	-0.82	0.57	0.01572
Rnf11	-0.57	0.68	0.01588
Gm20536	-0.55	0.68	0.01600
Plk2	-0.69	0.62	0.01602
Gm24262	1.19	2.29	0.01603
Krt8	-0.58	0.67	0.01604
Gm10717/Gm10715/Gm17535	0.67	1.59	0.01613
Lpl	-0.64	0.64	0.01618
Topbp1	-0.53	0.69	0.01624
Mir1948	1.01	2.01	0.01638
Gm13962	-0.61	0.65	0.01644
Tmem43	-0.71	0.61	0.01645
Mir669j	-0.59	0.67	0.01646
Mme	-0.83	0.56	0.01647
Cryl1	-0.72	0.61	0.01651
Rpl21-ps7	0.87	1.83	0.01663
mt-Tr	1.00	2.01	0.01664
Tmem159	-0.53	0.69	0.01665
Mknk2	-0.54	0.69	0.01665

Aldh3a2	-0.68	0.62	0.01672
Srxn1	-0.63	0.65	0.01676
Slco1a1	1.09	2.13	0.01679
Idh2	-0.60	0.66	0.01682
1810022K09Rik	-0.82	0.57	0.01687
Gm22685	1.25	2.38	0.01694
Otud7b	-0.63	0.65	0.01698
mt-Ts1	0.86	1.81	0.01705
Rhox2g	0.71	1.63	0.01712
Olfr205	-0.71	0.61	0.01727
Grpel2	-0.59	0.66	0.01734
Ighv5-6/Igh-V7183	0.72	1.65	0.01734
Prodh	0.66	1.58	0.01740
Mc1r	0.61	1.52	0.01807
Ighv1-7	-0.78	0.58	0.01816
Napepld	-0.56	0.68	0.01855
Fitm2	-0.58	0.67	0.01858
Mt1	-0.54	0.69	0.01878
Mir297a-4	0.88	1.84	0.01910
Abcc3	-0.52	0.70	0.01921
Cyp2c55	-0.66	0.63	0.01931
Car1	0.80	1.74	0.01944
Gm22260	0.61	1.53	0.01945
Entpd5	-0.59	0.66	0.01952
Postn	-0.67	0.63	0.01953
Cyp4a14	-1.75	0.30	0.01981
Mir5123	-0.81	0.57	0.01983
Rpl15-ps2	0.83	1.77	0.01987
Snord118	0.63	1.55	0.02028
Gm22723	0.72	1.65	0.02052
Tubb2a	-1.22	0.43	0.02068
Snora61	0.70	1.62	0.02089
1700019M22Rik	0.84	1.79	0.02103
Cpxm1	-0.61	0.65	0.02116
Cyp8b1	0.69	1.62	0.02117
C330024D21Rik	-0.55	0.69	0.02122
C7	-0.58	0.67	0.02138
Gm11168	0.64	1.56	0.02156
Gm25713	-0.58	0.67	0.02164

Sccpdh	-0.62	0.65	0.02180
Cd36	-0.84	0.56	0.02184
Gm24054	1.24	2.36	0.02189
Camk2a	0.81	1.76	0.02198
Gm14399	0.68	1.60	0.02204
Gm22866	1.01	2.01	0.02211
Ubd	-0.68	0.62	0.02218
Gm7789	0.70	1.62	0.02246
Klra14-ps	-0.91	0.53	0.02249
Rdh16	-0.67	0.63	0.02266
Cidea	-1.79	0.29	0.02284
Ly6g	-0.62	0.65	0.02290
Gm24949	0.78	1.72	0.02291
Gm6897	0.66	1.58	0.02293
Gm23927	0.87	1.83	0.02302
Acpp	0.83	1.78	0.02315
Mir151	-0.56	0.68	0.02327
Cyp3a11	-0.67	0.63	0.02345
Gm25521	-0.58	0.67	0.02356
Tmem81	-0.57	0.68	0.02360
Acaca	-0.65	0.64	0.02369
Gm24014	0.61	1.53	0.02384
Rpl32/Snora7a	0.66	1.58	0.02385
Purg	-0.54	0.69	0.02386
Rap1gap	-0.52	0.70	0.02432
Gm10719	0.67	1.59	0.02486
AI839979	-0.59	0.67	0.02490
Acta2	-0.64	0.64	0.02498
Clec7a	-0.56	0.68	0.02506
Gm21797	-0.63	0.64	0.02507
Akr1c18	-0.65	0.64	0.02522
Rnase2a	0.82	1.77	0.02526
Ect2	-0.56	0.68	0.02537
Sgol2a	-0.54	0.69	0.02545
Moxd1	2.92	7.58	0.02549
Slc17a4	-0.59	0.66	0.02586
Tff3	0.82	1.76	0.02590
Lum	-0.53	0.69	0.02596
Me1	-0.66	0.63	0.02638

Ces4a	0.95	1.93	0.02646
Mir466b-8/Mir466e	1.17	2.25	0.02649
Mir466b-8/Mir466e	1.17	2.25	0.02649
Vnn1	-0.82	0.57	0.02657
Snord47	0.67	1.59	0.02667
Krtap31-1	-0.53	0.69	0.02704
Olfr472	0.61	1.53	0.02724
Traj22	-0.52	0.70	0.02726
Il1b	-0.82	0.56	0.02740
n-R5s71	0.62	1.54	0.02787
Extl1	0.61	1.52	0.02849
1700113A16Rik	0.64	1.56	0.02849
Gm14406	-0.54	0.69	0.02864
Gm14403	0.89	1.85	0.02887
Gm14442	0.69	1.61	0.02912
Snord65	0.66	1.57	0.02924
Gm10719/Gm10721/Gm10718	0.70	1.62	0.02950
Nqo1	-0.71	0.61	0.02954
n-R5s80	0.64	1.56	0.02955
Olfr437	0.59	1.50	0.02958
Cxadr	-0.60	0.66	0.02969
Gm14405	0.72	1.65	0.02985
Gm20461	-0.64	0.64	0.03005
Gm15318/LOC102631757	0.89	1.85	0.03012
Gm24727	0.83	1.78	0.03021
Igkv8-30	0.97	1.96	0.03049
Rgs16	-0.54	0.69	0.03055
Gm23747	-0.68	0.62	0.03071
Gm13657	-0.70	0.61	0.03093
Sqstm1	-0.52	0.70	0.03155
Snord1b	1.24	2.35	0.03165
Ephx1	-0.56	0.68	0.03297
Gm23598	0.94	1.92	0.03314
Gm24897	0.63	1.55	0.03336
Snord13	0.62	1.54	0.03354
Npr2	0.64	1.56	0.03373
Vmn2r60	-0.58	0.67	0.03486
Scara5	0.65	1.57	0.03494
Gm24128	-1.20	0.43	0.03519

Gm25770	0.60	1.52	0.03641
Gm11963	0.67	1.59	0.03672
Robo1	-0.54	0.69	0.03709
Fermt1	-0.53	0.69	0.03757
S100a10	-0.62	0.65	0.03846
Gm10717	0.81	1.76	0.03883
Snord34/Rpl13a	0.90	1.86	0.04017
Gm22300	0.66	1.58	0.04025
Gm6929	0.61	1.53	0.04038
Igkv6-29	0.70	1.62	0.04059
Slc13a3	0.62	1.54	0.04074
Serpina4-ps1	1.04	2.05	0.04086
Shcbp1	-0.63	0.64	0.04112
Gna14	0.85	1.80	0.04112
Ncl/Snora75	0.91	1.88	0.04152
Gpr137b	-0.52	0.70	0.04237
Mtch2	-0.52	0.70	0.04294
Igkv6-17	-1.53	0.35	0.04299
Ighv5-4/Igh-VJ558	-0.74	0.60	0.04300
Trav9-4	0.66	1.58	0.04327
Olfr368	0.62	1.54	0.04366
Gm24519	0.87	1.82	0.04420
Arhgap19	-0.54	0.69	0.04460
Gm7809/LOC102633627	-0.61	0.66	0.04522
Gm7075	1.08	2.11	0.04525
Olfr1348	0.63	1.55	0.04535
Gm23723	-0.53	0.69	0.04542
Hibadh	0.64	1.56	0.04550
Olfr1388	-0.59	0.66	0.04606
Vmn1r157	-0.67	0.63	0.04610
Gm24984	-0.77	0.59	0.04619
Gm16299	0.62	1.54	0.04636
C4a	0.92	1.89	0.04648
Gm24861	-0.82	0.57	0.04664
Gm24869	0.72	1.65	0.04686
Gm26423	-0.63	0.65	0.04698
Bst2	-0.70	0.61	0.04698
Gm24112	-0.67	0.63	0.04704
Gm24405	0.67	1.59	0.04721

Mir3092	-0.52	0.70	0.04800
3000002C10Rik	0.59	1.50	0.04801
Mki67	-0.57	0.68	0.04825
Egr1	-0.74	0.60	0.04859
Sik1	-0.52	0.70	0.04860
Gm24494	0.82	1.77	0.04882
Olfr912	0.67	1.59	0.04916
A4gnt	-0.67	0.63	0.04969
Pgrmc2	-0.61	0.66	0.04970

Table S6. Lipid metabolism is inhibited in shRNA-ACOT1 mice, as identified by microarray analysis. In IPA, a z-score≤-2 corresponds to a highly inhibited process (shown in bold).

Diseases or Functions Annotation	p-value	Z-score
**Synthesis of lipid**	**1.08E-12**	**-3.05**
**Fatty acid metabolism**	**9.05E-20**	**-2.75**
**Metabolism of sphingolipid**	**1.84E-05**	**-2.49**
**Synthesis of sphingolipid**	**3.70E-06**	**-2.46**
**Proliferation of hepatic stellate cells**	**6.38E-03**	**-2.00**
Clearance of lipid	2.53E-05	-1.98
Activation of hepatic stellate cells	5.07E-02	-1.98
Concentration of choline-phospholipid	1.05E-06	-1.96
Synthesis of fatty acid	1.95E-06	-1.91

Table S7. Networks corresponding to DEGs between shRNA-ACOT1 mice and controls. Networks related to lipid metabolism are shown in Bold.

ID	Top Diseases and Functions	Score	Focus Molecules
**1**	**Lipid Metabolism, Small Molecule Biochemistry, Cell-To-Cell Signaling and Interaction**	**51**	**30**
2	Auditory Disease, Cellular Movement, Embryonic Development	36	24
**3**	**Lipid Metabolism, Small Molecule Biochemistry, Vitamin and Mineral Metabolism**	**34**	**23**
4	Connective Tissue Disorders, Developmental Disorder, Hereditary Disorder	30	21
5	Developmental Disorder, Hereditary Disorder, Immunological Disease	30	21
6	Cardiovascular System Development and Function, Cell Morphology, Cellular Development	28	20
7	Hematological Disease, Immunological Disease, Cancer	28	20
8	Energy Production, Lipid Metabolism, Small Molecule Biochemistry	26	19
9	Endocrine System Development and Function, Lipid Metabolism, Small Molecule Biochemistry	26	19
**10**	**Carbohydrate Metabolism, Lipid Metabolism, Molecular Transport**	**24**	**18**
11	Drug Metabolism, Glutathione Depletion in Liver, Molecular Transport	24	18
12	Cellular Development, Connective Tissue Development and Function, Tissue Development	22	17
**13**	**Lipid Metabolism, Small Molecule Biochemistry, Embryonic Development**	**22**	**17**
**14**	**Lipid Metabolism, Small Molecule Biochemistry, Nucleic Acid Metabolism**	**20**	**16**
15	Organismal Injury and Abnormalities, Renal Damage, Renal Tubule Injury	20	16
16	Post-Translational Modification, Protein Degradation, Protein Synthesis	20	16
17	Cell-mediated Immune Response, Cellular Development, Cellular Function and Maintenance	20	16
18	Developmental Disorder, Hereditary Disorder, Ophthalmic Disease	20	16
19	Humoral Immune Response, Protein Synthesis, Dermatological Diseases and Conditions	19	15
**20**	**Lipid Metabolism, Small Molecule Biochemistry, Cell Morphology**	**19**	**15**
21	Skeletal and Muscular System Development and Function, Cardiovascular Disease, Organismal Injury and Abnormalities	17	14
22	Dermatological Diseases and Conditions, Developmental Disorder, Hereditary Disorder	17	14
23	Cell Morphology, Connective Tissue Disorders, Dermatological Diseases and Conditions	17	14
**24**	**Lipid Metabolism, Molecular Transport, Small Molecule Biochemistry**	**15**	**13**
25	Carbohydrate Metabolism, Cancer, Organismal Injury and Abnormalities	14	12

Table S8: Differentially abundant lipids between ACOT1 overexpressing mice and ACOT1 shRNA mice. n= number of lipids.

Lipid	Name	ACOT1 vector UP (n)	shRNA-ACOT1 UP (n)	ACOT1 vector DOWN (n)	shRNA-ACOT1 DOWN (n)
**Glycerophospholipids**					
Fatty Acid	Fatty Acid	0	3	5	1
CL	Cardiolipin	7	10	3	0
DMPE	Dimethyl-phosphatidyl ethanolamine	7	1	1	3
LPE	Lyso-phosphatidyl ethanolamine	3	0	0	0
LPI	Lyso-phosphatidyl inositol	0	1	1	0
LPIP	Lyso-PI Triphosphate	0	1	0	0
LPIP3	Lyso-PI Triphosphate	0	1	0	1
LPS	Lyso-phosphatidyl serine	1	0	0	0
MMPE	Monomethyl-phosphatidyl ethanolamine	5	0	0	0
NAPE	N-Acyl-phosphatidyl ethanolamine	1	1	4	6
PA	Phosphatidic Acid	1	3	0	0
PC	Phosphatidyl choline	3	0	0	4
PE	Phosphatidyl ethanolamine	3	0	1	4
PG	Phosphatidyl glycerol	2	1	0	0
PI	Phosphatidyl inositol	1	1	0	0

PIP2	PI Diphosphate	0	1	0	0
PIP3	PI Triphosphate	0	1	0	0
PS	Phosphatidyl serine	1	2	3	5
**Sterol Lipids**					
CE	Cholesteryl Ester	1	1	0	0
**Glycerolipids**					
Fatty Acid	Fatty Acid	0	6	6	0
MADAG	Monoalkyl DAG	0	1	1	0
TAG	Tricylglyceri de	0	1	1	0
**Sphingolipids**					
CerP	Ceramide phosphate	0	0	2	0
GD2	Disialogangl ioside	0	3	0	0
GM1	Monosialotet rahexosylgan glioside	0	2	0	0
IPC	Inositol phosphorylc eramide	1	1	0	0
SM	Sphingomyel in	1	0	0	0

Supplemental Figures

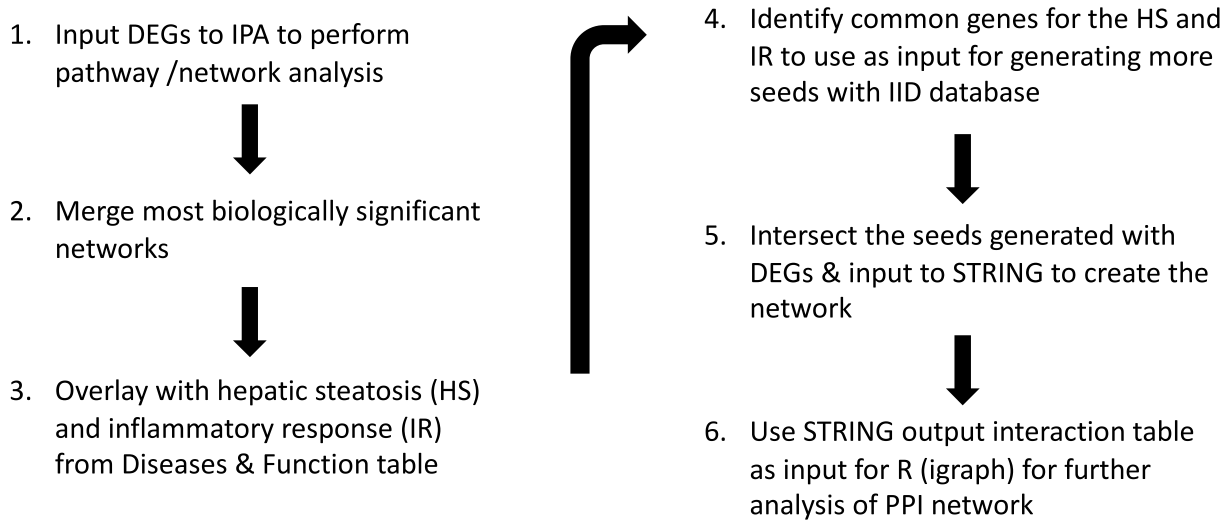

Figure S1. Workflow for analysis of network evolution from steatosis to MASH. (DEGs = Differentially Expressed Genes).

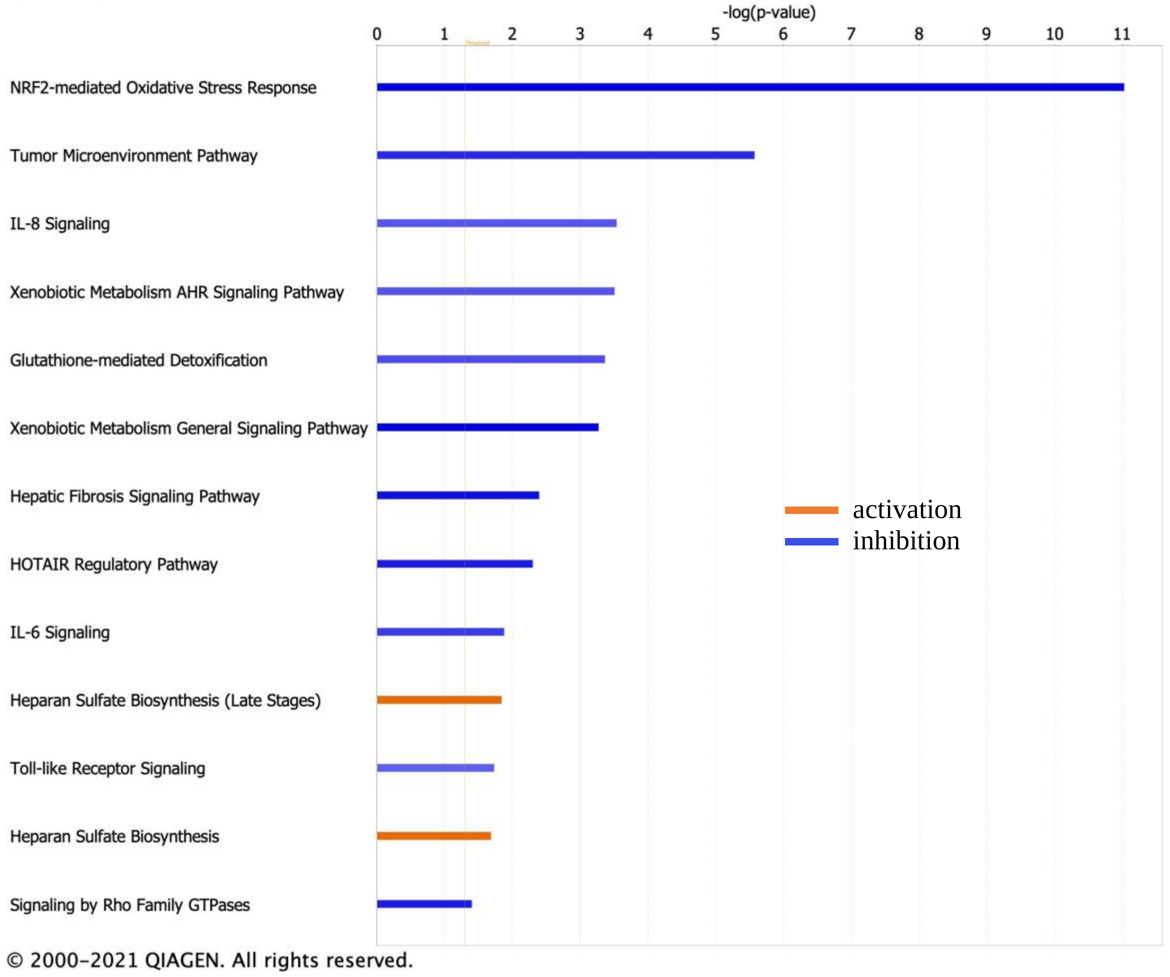

Figure S2. Canonical pathways significantly dysregulated as identified by IPA from shRNA-ACOT1 mice (n=3) vs control group (n=3) comparison.
